# Polyphasic Analysis Reveals Potential Petroleum Hydrocarbon Degradation and Biosurfactant Production by Rare Biosphere Thermophilic Bacteria From Deception Island, an Active Antarctic Volcano

**DOI:** 10.3389/fmicb.2022.885557

**Published:** 2022-05-04

**Authors:** Júnia Schultz, Isabella Campelo Vilardi Argentino, René Kallies, Ulisses Nunes da Rocha, Alexandre Soares Rosado

**Affiliations:** ^1^Microbial Ecogenomics and Biotechnology Laboratory, Biological and Environmental Science and Engineering Division, King Abdullah University of Science and Technology, Thuwal, Saudi Arabia; ^2^Red Sea Research Center, Biological and Environmental Science and Engineering Division, King Abdullah University of Science and Technology, Thuwal, Saudi Arabia; ^3^Laboratory of Molecular Microbial Ecology, Institute of Microbiology, Federal University of Rio de Janeiro, Rio de Janeiro, Brazil; ^4^Department of Environmental Microbiology, Helmholtz Centre for Environmental Research–UFZ, Leipzig, Germany; ^5^Bioscience Program, Biological and Environmental Science and Engineering Division, King Abdullah University of Science and Technology, Thuwal, Saudi Arabia

**Keywords:** extremophiles, thermophiles, polar volcano, Antarctica, biosurfactants, oil degradation, bacterial isolation

## Abstract

Extreme temperature gradients in polar volcanoes are capable of selecting different types of extremophiles. Deception Island is a marine stratovolcano located in maritime Antarctica. The volcano has pronounced temperature gradients over very short distances, from as high as 100°C in the fumaroles to subzero next to the glaciers. These characteristics make Deception a promising source of a variety of bioproducts for use in different biotechnological areas. In this study, we isolated thermophilic bacteria from sediments in fumaroles at two geothermal sites on Deception Island with temperatures between 50 and 100°C, to evaluate the potential capacity of these bacteria to degrade petroleum hydrocarbons and produce biosurfactants under thermophilic conditions. We isolated 126 thermophilic bacterial strains and identified them molecularly as members of genera *Geobacillus, Anoxybacillus*, and *Brevibacillus* (all in phylum Firmicutes). Seventy-six strains grew in a culture medium supplemented with crude oil as the only carbon source, and 30 of them showed particularly good results for oil degradation. Of 50 strains tested for biosurfactant production, 13 showed good results, with an emulsification index of 50% or higher of a petroleum hydrocarbon source (crude oil and diesel), emulsification stability at 100°C, and positive results in drop-collapse, oil spreading, and hemolytic activity tests. Four of these isolates showed great capability of degrade crude oil: FB2_38 (*Geobacillus*), FB3_54 (*Geobacillus*), FB4_88 (*Anoxybacillus*), and WB1_122 (*Geobacillus*). Genomic analysis of the oil-degrading and biosurfactant-producer strain FB4_88 identified it as *Anoxybacillus flavithermus*, with a high genetic and functional diversity potential for biotechnological applications. These initial culturomic and genomic data suggest that thermophilic bacteria from this Antarctic volcano have potential applications in the petroleum industry, for bioremediation in extreme environments and for microbial enhanced oil recovery (MEOR) in reservoirs. In addition, recovery of small-subunit rRNA from metagenomes of Deception Island showed that Firmicutes is not among the dominant phyla, indicating that these low-abundance microorganisms may be important for hydrocarbon degradation and biosurfactant production in the Deception Island volcanic sediments.

## Introduction

Antarctica is a mosaic of extreme, mainly cold ecosystems. However, the continent has four active volcanoes, three on the continent itself (Mounts Erebus, Melbourne, and Rittmann) and one in maritime Antarctica (Deception Island) (Herbold et al., 2014). These are unique volcano habitats; in particular, Deception Island, a stratovolcano with fumarolic emissions, thermal springs, and hot soils that can reach temperatures as high as 110°C, is under marine influence (Caselli et al., 2004). These characteristics provide ideal conditions and opportunities to select a versatile and extremely diverse community of microorganisms adapted to psychrophilic and thermophilic stresses.

Although the microbial diversity of Deception Island has been studied in recent decades, knowledge of these microbes is still in its infancy. Previous studies found culturable thermophilic bacteria belonging to the genera *Geobacillus, Bacillus, Brevibacillus*, and *Thermus* (Muñoz et al., 2011; Bendia et al., 2018a). Several DNA sequences of extremophiles (psychrophiles, thermophiles, and hyperthermophiles) have been evaluated, using metagenomic analyses and the 16S rRNA gene. Bendia et al. (2018b) found a prevalence of the phylum Proteobacteria (43.75%), followed by Bacteroidetes (17.11%) along environmental gradients on Deception Island. Phylum Firmicutes was also present, with a relative abundance of 0.38%.

Metagenomic approaches, in which the total DNA is extracted directly from environmental samples and shotgun-sequenced (Kunin et al., 2008), have been used to investigate microbial communities and their functions in various extreme environments, such as thermal pools (Wilkins et al., 2019), desert biological soil crusts (Meier et al., 2021), permafrost (Woodcroft et al., 2018), and Antarctic microbial mat and soil (Zaikova et al., 2019; Bendia et al., 2021). Nearly complete genomes have been assembled from shotgun-sequenced metagenomes from these environments. However, it has proven difficult to assemble genomes from populations with relative abundances below 1% by using current metagenome sequencing and assembly approaches (Albertsen et al., 2013). Therefore, only genomes of the dominant populations are recovered from a simple microbial community (Somerville et al., 2019).

Despite the advent of omics analysis, culture studies are still important to assess the physiological properties of bacterial cells and to cover the limits of detection of independent-culture methods (Zamkovaya et al., 2021). Such studies allow us to understand the survival strategies and metabolic adaptations of bacteria (Bendia et al., 2021) and to explore their potential for use in different areas of biotechnology, through culturomics and deep genomic analyses (Schultz and Rosado, 2019, 2020).

Bacterial thermophiles are a source of a variety of bioproducts and can be isolated from different environments, such as geothermal sites in Antarctica. To date, only a few studies have attempted to isolate thermophilic microorganisms from Deception Island for potential use in industrial processes. Cultured bacterial isolates belonging to *Geobacillus, Bacillus, Brevibacillus*, and *Thermus* were explored and showed positives results for the production of lipases (Muñoz et al., 2013, 2015). Flores et al. (2018) partially characterized a thermophilic microorganism identified as *Bacillus gelatini*, which showed stable glutamate dehydrogenase activity at high temperatures. Additionally, a new thermophile identified as *Albidovulum* sp. SLM16 showed amine transaminase activity (Márquez and Blamey, 2019). Remarkably, most of the isolated strains have been assigned to Firmicutes, a taxon found in low abundance on Deception Island (Bendia et al., 2018b). Increasing research on the rare biosphere is expanding our understanding of the importance of these less abundant organisms in ecology and biotechnology (Jousset et al., 2017). Rare microbial communities are described as potential sources for new genes, pathways, and solutions for environmental decontamination to apply in bioremediation and bioprospecting (Pascoal et al., 2020).

Geothermal environments in Antarctica, including Deception Island, are poorly explored for biotechnologically useful bioproducts. Discovery and study of new microorganisms, particularly thermophiles, from this extreme continent are important not only for the contribution to knowledge of microbial ecology and biodiversity but as a source of novel biocompounds with potential uses for industrial, environmental, medical, and commercial purposes (Perfumo et al., 2018). Because of their thermal and chemical stability as well as structural and molecular modifications, thermophiles can be used for processes that require high temperatures, such as in the bioenergy, bioconversion, pharmaceutical, biomedical, detergent, agriculture, and oil industries (Schultz and Rosado, 2020; Zuliani et al., 2021). In the oil industry, thermophiles have received increasing attention for two main applications: microbial enhanced oil recovery (MEOR), a tertiary oil recovery technique (Datta et al., 2022), and bioremediation of extreme environments contaminated by crude oil and its derivatives (Almeida et al., 2016; Schultz and Rosado, 2020).

The negative impacts of human activities across Antarctica, including Deception Island, caused by a distinct set of threats over and above those associated with global climate change, such as tourism, cargo, structures and facilities, and spills of oil and its derivatives on land and in marine environments, significantly affect the wildlife and disturb the polar ecosystem (Chown and Brooks, 2019; Convey and Peck, 2019). Bioremediation processes hold great promise for exploiting the ability of polar microbes to metabolize hydrocarbon substrates. For bioremediation strategies, it is first necessary to determine the structure and diversity of the microbial community in the contaminated site capable of degrading petroleum hydrocarbons, because the effectiveness of bioremediation will depend on the functionality of indigenous microorganisms in degrading the oil compounds (Goswami et al., 2018; Sharma and Pandey, 2021). The hydrophobicity generally limits the rate of biodegradation; however, microbes can produce biosurfactants and bioemulsifiers, amphipathic organic molecules with tensioactive and emulsification effects, which enhance their access to target hydrocarbon substrates and bioavailability of the compound (Pacwa-Płociniczak et al., 2011; Sharma et al., 2022).

Extreme biosurfactants and bioemulsifiers produced by thermophiles remain little explored for biotechnological purposes. Because the compounds and the microbial-producer remain active and stable in high temperatures, such as found in oil reservoirs and geothermal sites, they can be potentially applied in MEOR and also in bioremediation of hot environments (Dhanarajan et al., 2017; Geetha et al., 2018; Schultz and Rosado, 2020). The momentum is increasing to bioprospect in more hostile environments, such as geothermal environments in Antarctica, that might be a source of novel and more efficient products.

We hypothesized that bacteria isolated from thermophilic Antarctic sediments would contain as-yet-undescribed and biotechnologically important metabolic capacities for oil degradation and biosurfactant production that are screenable in tests and have potential applications in the oil industry. To test this hypothesis, we isolated thermophilic bacteria from sediments associated with fumaroles at two geothermal sites on Deception Island, at temperatures between 50 and 100°C, to evaluate the potential capacity of these bacteria to degrade petroleum hydrocarbons and produce biosurfactants and/or bioemulsifiers under thermophilic conditions. We related the phylogeny of the isolates recovered in this study to those of the reconstructed small-subunit (SSU) rRNA gene sequences, to determine what fraction of the bacterial diversity was recovered by isolation using simple modifications to existing culture methods. Finally, we assessed metabolic functions related mainly to hydrocarbon degradation and biosurfactant and bioemulsifier production, through genomic exploration.

## Materials and Methods

### Study Site and Sampling Strategy

Sampling was performed during the XXXIV Brazilian Antarctic Expedition (December 2015–January 2016) on the volcanic Deception Island, Antarctica, at the geothermally active sites of Whalers Bay (WB) (–62.979611, –60.555778) and Fumarole Bay (FB) (–62.967417, –60.710111). In Whalers Bay, samples were collected in fumaroles with temperatures of 50°C (WB1) and 60°C (WB2). In Fumarole Bay, we collected samples in fumaroles with temperatures of 55°C (FB1), 70°C (FB2), 80°C (FB3), and 100°C (FB4). The fumaroles were less than 2 m apart, and the Whalers Bay and Fumarole Bay transects were approximately 10 km apart. All fumaroles were in the intertidal zone, except for a fumarole with a temperature of 80°C from Fumarole Bay (FB3), which was in the subtidal zone (50 cm below the water surface). Approximately 500 g of sediment was collected from each sampling point, from a depth of 0 to 5 cm. The samples were placed in sterile plastic bags and immediately transferred to the laboratory, where they were stored at 4°C until arrival at the Federal University of Rio de Janeiro, Brazil, in April 2016.

### Isolation of Thermophiles From Environmental Samples

A summary of the experimental design is available in Supplementary Figure 1. To isolate thermophiles from the fumaroles on Deception Island, we inoculated the samples into six culture media at 55°C growth temperature. The temperature of 55°C was selected based on the temperature found in Fumarole Bay (55–100°C) and Whalers Bay (50–60°C). First, each sediment sample was homogenized and 10 g was added to 90 mL of saline solution (0.85%) with glass beads and agitated for 2 h. Serial tenfold dilutions (10^–1^–10^–3^) were prepared in the same diluent, and in triplicate, 0.1 mL of each dilution was spread over the surface of Petri dishes containing six different culture media. The media were lysogeny agar (Bertani, 1951), marine agar (Zobell, 1941), glucose yeast malt (Mendez et al., 1985), DSMZ 260 medium (DSMZ GmbH), calcium phytate medium (Richardson and Simpson, 2011), and NBRIP (National Botanical Research Institute Phosphate) medium (Nautiyal, 1999). Plates were incubated at 55°C for 48 h. The composition of the used culture media is available in Supplementary Table 1.

After the incubation period, we randomly selected three colonies of each culture for the isolation procedure. A total of 126 colonies were successfully isolated and then selected for subsequent molecular analyses. The pure strains were stored at -80°C with 20% (v/v) glycerol and deposited in the Culture Collection of the Microbial Molecular Ecology Laboratory, Federal University of Rio de Janeiro.

### 16S rRNA Sequence Analysis and Identification

Samples were cultured in lysogeny broth for 48 h at 55°C with constant shaking, and bacterial genomic DNA was extracted using the Wizard ^®^ Genomic DNA Purification Kit (Promega, United States) according to the manufacturer’s instructions. Isolates were identified based on partial sequencing of the 16S rRNA gene for molecular identification. An approximately 1450-bp fragment of the 16S rRNA gene was amplified, using universal primers 27F (5′-AGA-GTT-TGA-TCM-TGG-CTC-AG-3′) and 1492R (5′-TAC-GGY-TAC-CTT-GTT-ACG-ACT-T-3′) (Lane, 1991) with a PCR reaction, which consisted of 10 pM of each primer, 10 ng of DNA, BSA (1:20), MyTaq ^®^ (Bioline, United States), and 2X buffer containing dNTPs and MgCl_2_ (Bioline, United States) for a total volume of 25 μL. The amplification was performed in a thermocycler, with an initial denaturation at 95°C for 3 min, followed by 25 cycles of 95°C for 30 s, 55°C for 30 s, and 72°C for 30 s, with a final extension at 72°C for 5 min. PCR products were purified with SureClean Plus (Bioline, United States), quantified by Qubit 4.0 with the Qubit ^®^ dsDNA HS Assay Kit (Life Technologies, United States), and examined by electrophoresis on 1.5% agarose gels stained with ethidium bromide and observed under UV light. After purification, approximately 25 ng of the amplicons from each isolate was sent for sequencing by Sanger’s chain termination technique.

The sequences were initially analyzed using Geneious Prime v. 2022.0.2^1^ to check for quality and treated, and contigs were formed from the overlap of the amplified sequences with the two primers.

### Metagenomic Sequencing and Reconstruction of Small-Subunit of rRNA Genes From Metagenome Reads and Comparisons of Small-Subunit rRNA Genes From Isolates

The DNA used to perform the metagenomic sequencing was extracted from samples collected in the same area where the samples for isolating bacteria were obtained. The DNA extraction and metagenome sequencing (Shotgun) methods were described by Bendia et al. (2021). Reads were filtered using SICKLE software (Joshi and Fass, 2011), using a minimum Phred score of 30. Metagenomics RAST server (MG-RAST) provides sequence quality control by removing duplicate sequences and selecting sequences larger than 80 bp and a Phred score greater than 30 for analysis (Meyer et al., 2008). All the metagenomic sequences were retrieved from the MG-RAST server (project ID mgp15628).

Nearly full-length SSU rRNA sequences were reconstructed from metagenome sequences with MATAM (Mapping-Assisted Targeted-Assembly for Metagenomics; Pericard et al., 2018). We used Trimmomatic (Bolger et al., 2014) as a sequence quality trimmer. Paired-end reads where both reads were at least 60 nucleotides in length after trimming were used as inputs. The reads were mapped to release 132 of the Silva SSU Ref NR database (Quast et al., 2013), clustered at 97% similarity, using the tool SortMeRNA (Kopylova et al., 2012). To the SILVA database v.132, we added the SSU rRNA gene sequences of the isolates cultured in this study. Chimeric sequences were removed by UCHIME (Edgar et al., 2011) implemented in VSEARCH (Rognes et al., 2016) and querying the Silva 132 SSU Ref Nr99 database.

We also attempted to determine the bacterial fraction of the metagenomes that were also recovered by culturing. We clustered sequences with more than 97% similarity, defined here as the same Operational Taxonomic Unit (OTU). To determine which sequences were more than 97% similar, we used the tool UCLUST from the USEARCH package (Edgar, 2010), where we clustered the SSU rRNA gene sequences of all isolates with those of the SSU rRNA genes reconstructed from metagenomes. Briefly, the fasta files containing the sequences of all isolates were concatenated into the fasta files containing the ribosomal sequences reconstructed by MATAM. Sequences were then sorted by length prior to OTU clustering using UCLUST.

### Screening of Oil-Degrading Thermophilic Bacteria

We randomly selected 100 isolates previously identified from the 16S rRNA gene to test their capacity to degrade petroleum hydrocarbons. We reactivated the isolates in LB broth at 55°C for 48 h with constant shaking, and then transferred a total volume of 100 μL of each culture to plates containing solid Bushnell-Haas (BH) medium (0.02 g L^–1^ CaCl_2_ × 2 H_2_O, 0.2 g L^–1^ MgSO_4_ × 7 H_2_O, 1 g L^–1^ K_2_HPO_4_, 1 g L^–1^ KH_2_PO_4_, 1 g L^–1^ NH_4_NO_3_, 0.05 g L^–1^ FeCl_3_ × 2 H_2_O, agar 1 g L^–1)^ supplemented with 2% crude oil (Atlas, 1995). Isolates that grew on plates with BH supplemented with oil were enriched in BH broth supplemented with crude oil (2%) (Cury et al., 2015; de Jesus et al., 2015) at 55°C for 48 h and submitted to the oil-drop test in well-plates, as described by Youssef et al. (2004), as a qualitative test of degradation (Supplementary Figure 1).

Briefly, the cells were centrifuged and resuspended in saline solution (0.85%) to remove any remaining carbon source from the medium. Cells were resuspended in 5 mL of saline and inoculated into 24-well plates, where each well received 2 mL of BH broth, 20 μL crude oil, and 160 μL bacterial culture of each isolate. All inoculations were performed in triplicate, including a negative control (BH broth with oil and deionized water, without bacterial inoculation) and a positive control (oil-degrading strain D24M), incubated at 55°C, and observed for up to 21 days. Results were considered positive when the oil changed in appearance as visually compared with the controls.

### Screening for Biosurfactant and Bioemulsifier Production

To determine the capacity of these thermophilic oil-degrading bacteria to produce biosurfactants, 50 isolates were randomly selected, based on the positive results identifying them as oil-degraders. Bacteria were grown aerobically in 250-mL Erlenmeyer flasks with 100 mL of BH broth supplemented with 2% yeast extract, and were maintained at 55°C for 7 days with constant shaking (165 rpm) (Mani et al., 2016). After incubation, the bacteria culture from each flask was centrifuged for 15 min at 13,000 × *g* and this cell-free culture broth was used for the screening assays. All screening experiments were performed in triplicate and the results expressed as the mean of three values.

#### Drop-Collapse Test

The drop-collapse test followed the protocol proposed by Jain et al. (1991). A drop of crude oil was pipetted onto a glass slide and 10 μL of the bacterial culture supernatant was added to the oil surface. The same was performed for the controls (SDS 1% and deionized water). If the oil drop became flat one min after the supernatant was added, the result was taken to be positive; otherwise, it was scored as negative.

#### Oil-Spreading Assay

As described by Morikawa et al. (2000), 10 μL of oil was added to a Petri dish containing 40 mL of deionized water, forming a thin layer of oil. Then, 10 μL of the supernatant from each isolate and the positive (1% SDS) and negative (deionized water) controls was added to the center of the oil layer. The diameter of the clear zone on the oil surface was measured and compared with the clear zones in the negative and positive controls.

#### Hemolysis Test

The hemolysis test was carried out as described by Mulligan et al. (1984): 10 μL of the supernatant culture and controls (1% SDS as a positive control and deionized water as a negative control) was inoculated as spots in Petri dishes containing blood-agar medium (5% defibrinated sheep’s blood; Laborclin ^®^). The plates were incubated for 48 h at 55°C; after the incubation period, if a clear halo (hemolysis zone) was present, the result was considered positive for biosurfactant production.

#### Emulsification Assay

The emulsification assay was performed according to Cooper and Goldenberg (1987), using crude oil and diesel. Cell-free culture broth (200 μL) was transferred to a glass tube containing 600 μL of deionized water and 1.2 mL of crude oil or diesel. The supernatant-water-oil mixture was agitated for 2 min in a vortex mixer and the results were analyzed after 24 h, applying the emulsification index calculation: EI (%) = (height of the emulsion layer/total height) × 100. The same procedure was performed with the controls (1% SDS and deionized water).

The resistance and stability of the biosurfactant when heated were evaluated by heating the supernatant at 100°C for 15 min (Couto et al., 2015) and assaying the emulsification of crude oil and diesel, as previously described.

### Genomic Sequencing and Data Analysis

To better understand the taxonomy and functionalities of thermophiles in cold environments, one bacterial strain (FB4_88) capable of degrading oil hydrocarbon and producing biosurfactant was selected for whole-genome sequencing and deep characterization of its genome.

#### Whole Genome Sequencing

An amount of 5 μg/μL of gDNA was used to construct paired-end sequencing libraries (2 × 150 bp) of 450-bp inserts, following the manufacturer’s protocol for the NEBNext ^®^ Fast DNA Fragmentation and Library Preparation Kit (New England Biolabs Inc.). Quality analysis of the final libraries was performed with an Agilent 2100 bioanalyzer (Agilent Technologies). The bacterial sample were sequenced on Illumina Hi-Seq 2500 platform, as recommended by the manufacturer.

#### Assembly, Gene Prediction, and Functional Annotation

For genome assembly, raw reads were quality-checked through FastQC (Andrews, 2010) and trimmed with Adapter Removal (Lindgreen, 2012) software. The estimated best k-mers were selected by KmerStream (Melsted and Halldórsson, 2014), followed by assembly using Edena (Hernandez et al., 2008) and SPAdes (Bankevich et al., 2012). The results were combined and the package PSI-CD-HIT (Fu et al., 2012), was used to remove the redundant contigs, producing a final contigs file. The quality of each assembly was checked in QUAST. Default parameters were used for all software, and the bacterial genomes were annotated with Prokka (Seemann, 2014).

The predicted contigs were analyzed with GO FEAT (Araujo et al., 2018), an on-line platform for functional annotation and enrichment of genomic data integrated with the UNIPROT,^2^ INTERPRO,^3^ PFAM,^4^ NCBI,^5^ and KEGG^6^ databases. Functional annotations were performed using Rapid Annotation of microbial genomes, using Subsystems Technology (RAST) (Aziz et al., 2008), and the results were used to infer functions at subsystem levels. BioSurfDB, an online server with a specific database for biodegradation, was used to predict genes related to hydrocarbon degradation and biosurfactant production. To predict biosynthetic gene clusters (BGCs) and bacteriocins, the web-based platforms antiSMASH 5.0 (Blin et al., 2021) and BAGEL 4.0 (van Heel et al., 2018) were used. Prophage sequences were identified using Phage Search Tool Enhanced Release (PHASTER) (Arndt et al., 2016).

#### Phylogenomic and Comparative Genomic Analyses

Phylogenomic analyses were conducted using JSpeciesW with default parameters. ANIb, ANIm, and TETRA values were calculated based on the genomes analyzed in this study and genomes of the family Bacillaceae available in public databases. Using the R project and the RStudio development environment, we combined and analyzed the results from ANIb. The relationship among the genomes was calculated using the dist function, using Euclidean distance, followed by the hclust function with the “average” method to cluster the results. The ggplot2 package was used to generate the scatter plot.

Subsequently, using the OrthoVenn2 platform with default parameters (Xu et al., 2019), orthologous groups were searched among the genome analyzed and the genomes from the public database (*n* = 6). The orthologous groups were plotted, displaying the number of common and exclusive orthologous groups of proteins present in the seven genomes.

BLAST Ring Image Generator (BRIG) (Alikhan et al., 2011) was used to provide a visual overview of the relationship between the analyzed genome and the genomes from a public database.

#### Data Availability

All Sanger sequences were deposited in GenBank (National Center for Biotechnology Information) in the Bioproject PRJNA554144, under accession numbers OM842843-OM842968. Metagenomic sequences are available in the MG-RAST server under the project ID mgp15628. The genome shotgun project for strain FB4_88 has been deposited at NCBI/GenBank under accession number JAKNCX000000000, in the Bioproject PRJNA554144. The version described here is JAKNCX000000000.

## Results

### Thermophiles Isolated From Deception Island and Phylogenetic Classification

We attempted to purify 245 thermophilic bacterial strains grown at 55°C from all media. Of these, 126 were successfully purified to single colonies, isolated from two geothermal environments on Deception Island: 32 strains from Whalers Bay and 94 strains from Fumarole Bay. The largest number of strains (45 isolates) were recovered from FB4, a Fumarole Bay sample, where the environmental temperature was 100°C (Supplementary Table 2).

**FIGURE 1:**
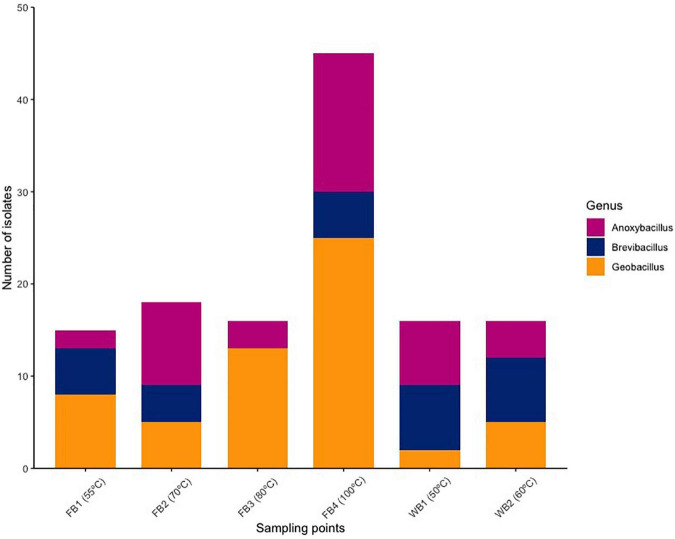
Relative abundances of the taxonomic groups assigned to the isolates grown at 55°C, at the maximum classification level. Environmental temperatures of each sample are shown. Sequences were assigned with 97% similarity against the SILVA database v. 132.

Firmicutes was the only bacterial phylum with which the sequences of the 16S rRNA gene of 126 isolates were affiliated. Most isolates were assigned to *Geobacillus* (46%, 58 isolates), followed by *Anoxybacillus* (31.8%, 39 isolates), *Brevibacillus* (22.2%, 28 isolates). *Geobacillus*-related isolates were found at every collection site and predominated in the fumaroles above 80°C. However, *Brevibacillus* was not isolated from FB3 (80°C), but was prominent in samples from Whalers Bay (WB1 and WB2) (Figure 1 and Supplementary Table 2).

### Comparison Between Isolates and Metagenomes

**FIGURE 2:**
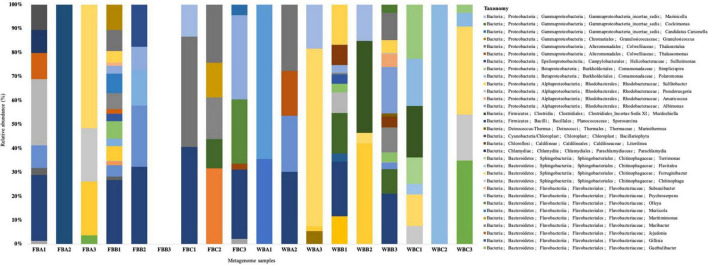
Relative abundances of the taxonomic groups assigned to the SSU rRNA, reconstructed from metagenomes.

To determine the fraction of the total phylotype diversity (OTUs weighted by relative abundance in the community) in the metagenomes from Deception Island recovered as isolates, we determined the overlap between sequences obtained from isolates and OTUs derived from sequences obtained from the metagenome. We recovered 52 rRNA 16S from the metagenomes (no assignment were observed in FFB3 sample) (Figure 2), however, no sequence corresponded to the genera isolated by culture-dependent methods from the sediment samples; only two genera from the Firmicutes phylum were retrieved (*Sporosarcina* and *Murdochiella*). This result indicates that the cultured isolates were not dominant in the samples and that the culture methods that we used selected specific groups of the bacterial community.

#### Screening for Oil-Degrading Bacteria and Biosurfactant/Bioemulsifier Producers

**FIGURE 3:**
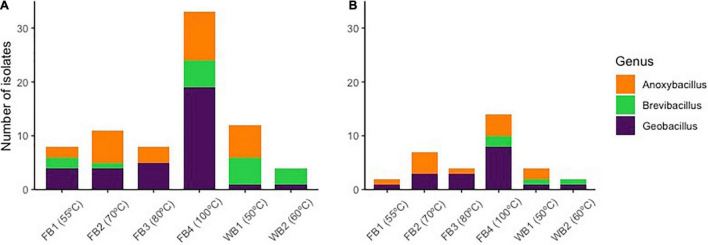
Relative abundances of the taxonomic groups assigned to the isolates grown at 55°C, at the maximum classification level, capable of degrading oil **(A)** and producing biosurfactants **(B)**. Environmental temperatures of each sample are shown. Sequences were assigned with 97% similarity against the SILVA database v. 132.

Many of the thermophilic isolates showed a high capacity to use crude oil as a carbon source. Of the 100 strains tested, a total of 76 isolates were grown in culture medium containing oil as the only carbon source, showing potential for the degradation of petroleum hydrocarbons (Table 1). In the drop test, 30 of the 76 isolates showed good results for degradation of crude oil to use as carbon source when compared visually with the controls. Of the nine strains that showed particularly good results, six were related to *Geobacillus*, from Fumarole Bay (FB2_38, FB3_47, FB3_50, FB4_79, FB4_85, and FB4_93) and Whalers Bay (WB1_122), and two isolates belonged to *Anoxybacillus* (FB4_88 and WB1_125) (Figure 3).

**TABLE 1:** List of thermophiles from Deception Island, Antarctica, capable of degrading crude oil and use as a carbon source, and their molecular identification.

Isolate code	Growth in crude oil	Oil drop test (triplicate)	SILVA v. 132 classification
**FB1_1**	Yes	+/–/–	Bacteria; Firmicutes; Bacilli; Bacillales; Bacillaceae; *Anoxybacillus*
FB1_2	Yes	–/–/–	Bacteria; Firmicutes; Bacilli; Bacillales; Bacillaceae; *Brevibacillus*
**FB1_3**	Yes	++/+/++	Bacteria; Firmicutes; Bacilli; Bacillales; Bacillaceae; *Anoxybacillus*
FB1_6	No	n.t.	Bacteria; Firmicutes; Bacilli; Bacillales; Bacillaceae; *Brevibacillus*
FB1_7	Yes	+/–/–	Bacteria; Firmicutes; Bacilli; Bacillales; Bacillaceae; *Brevibacillus*
FB1_8	Yes	+/–/–	Bacteria; Firmicutes; Bacilli; Bacillales; Bacillaceae; *Geobacillus*
FB1_9	No	n.t.	Bacteria; Firmicutes; Bacilli; Bacillales; Bacillaceae; *Geobacillus*
FB1_10	No	n.t.	Bacteria; Firmicutes; Bacilli; Bacillales; Bacillaceae; *Geobacillus*
**FB1_12**	Yes	+/+/+	Bacteria; Firmicutes; Bacilli; Bacillales; Bacillaceae; *Geobacillus*
**FB1_13**	Yes	+/+/++	Bacteria; Firmicutes; Bacilli; Bacillales; Bacillaceae; *Geobacillus*
**FB1_14**	Yes	+/+/+	Bacteria; Firmicutes; Bacilli; Bacillales; Bacillaceae; *Geobacillus*
FB2_21	No	n.t.	Bacteria; Firmicutes; Bacilli; Bacillales; Bacillaceae; *Anoxybacillus*
FB2_22	No	n.t.	Bacteria; Firmicutes; Bacilli; Bacillales; Bacillaceae; *Anoxybacillus*
FB2_23	Yes	+/–/–	Bacteria; Firmicutes; Bacilli; Bacillales; Bacillaceae; *Anoxybacillus*
**FB2_25**	Yes	–/–/–	Bacteria; Firmicutes; Bacilli; Bacillales; Bacillaceae; *Anoxybacillus*
**FB2_26**	Yes	+/–/–	Bacteria; Firmicutes; Bacilli; Bacillales; Bacillaceae; *Anoxybacillus*
**FB2_27**	Yes	+/+/+	Bacteria; Firmicutes; Bacilli; Bacillales; Bacillaceae; *Anoxybacillus*
**FB2_29**	Yes	+/–/–	Bacteria; Firmicutes; Bacilli; Bacillales; Bacillaceae; *Anoxybacillus*
FB2_30	No	n.t.	Bacteria; Firmicutes; Bacilli; Bacillales; Bacillaceae; *Brevibacillus*
**FB2_31**	Yes	+/+/+	Bacteria; Firmicutes; Bacilli; Bacillales; Bacillaceae; *Anoxybacillus*
FB2_32	Yes	+/–/–	Bacteria; Firmicutes; Bacilli; Bacillales; Bacillaceae; *Brevibacillus*
FB2_33	No	n.t.	Bacteria; Firmicutes; Bacilli; Bacillales; Bacillaceae; *Brevibacillus*
**FB2_35**	Yes	+/–/+	Bacteria; Firmicutes; Bacilli; Bacillales; Bacillaceae; *Geobacillus*
**FB2_36**	Yes	++/+/++	Bacteria; Firmicutes; Bacilli; Bacillales; Bacillaceae; *Geobacillus*
FB2_37	Yes	+/–/–	Bacteria; Firmicutes; Bacilli; Bacillales; Bacillaceae; *Geobacillus*
**FB2_38**	Yes	+++/++/++	Bacteria; Firmicutes; Bacilli; Bacillales; Bacillaceae; *Geobacillus*
FB2_39	No	n.t.	Bacteria; Firmicutes; Bacilli; Bacillales; Bacillaceae; *Brevibacillus*
FB3_43	Yes	+/–/–	Bacteria; Firmicutes; Bacilli; Bacillales; Bacillaceae; *Anoxybacillus*
**FB3_44**	Yes	+/+/+	Bacteria; Firmicutes; Bacilli; Bacillales; Bacillaceae; *Anoxybacillus*
FB3_45	Yes	+/–/–	Bacteria; Firmicutes; Bacilli; Bacillales; Bacillaceae; *Anoxybacillus*
FB3_46	No	n.t.	Bacteria; Firmicutes; Bacilli; Bacillales; Bacillaceae; *Geobacillus*
**FB3_47**	Yes	++/++/+++	Bacteria; Firmicutes; Bacilli; Bacillales; Bacillaceae; *Geobacillus*
FB3_48	No	n.t.	Bacteria; Firmicutes; Bacilli; Bacillales; Bacillaceae; *Geobacillus*
**FB3_49**	Yes	+/–/–	Bacteria; Firmicutes; Bacilli; Bacillales; Bacillaceae; *Geobacillus*
**FB3_50**	Yes	++/++/+++	Bacteria; Firmicutes; Bacilli; Bacillales; Bacillaceae; *Geobacillus*
FB3_51	No	n.t.	Bacteria; Firmicutes; Bacilli; Bacillales; Bacillaceae; *Geobacillus*
**FB3_54**	Yes	+++/–/+++	Bacteria; Firmicutes; Bacilli; Bacillales; Bacillaceae; *Geobacillus*
FB3_55	No	n.t.	Bacteria; Firmicutes; Bacilli; Bacillales; Bacillaceae; *Geobacillus*
**FB3_58**	Yes	+/–/–	Bacteria; Firmicutes; Bacilli; Bacillales; Bacillaceae; *Geobacillus*
FB4_62	No	n.t.	Bacteria; Firmicutes; Bacilli; Bacillales; Bacillaceae; *Anoxybacillus*
FB4_63	No	n.t.	Bacteria; Firmicutes; Bacilli; Bacillales; Bacillaceae; *Anoxybacillus*
FB4_64	No	n.t.	Bacteria; Firmicutes; Bacilli; Bacillales; Bacillaceae; *Anoxybacillus*
FB4_65	No	n.t.	Bacteria; Firmicutes; Bacilli; Bacillales; Bacillaceae; *Anoxybacillus*
**FB4_66**	Yes	+/+/+	Bacteria; Firmicutes; Bacilli; Bacillales; Bacillaceae; *Brevibacillus*
**FB4_67**	Yes	+/+/+	Bacteria; Firmicutes; Bacilli; Bacillales; Bacillaceae; *Anoxybacillus*
**FB4_68**	Yes	+/+/+	Bacteria; Firmicutes; Bacilli; Bacillales; Bacillaceae; *Anoxybacillus*
**FB4_69**	Yes	+/–/–	Bacteria; Firmicutes; Bacilli; Bacillales; Bacillaceae; *Anoxybacillus*
**FB4_70**	Yes	+/–/+	Bacteria; Firmicutes; Bacilli; Bacillales; Bacillaceae; *Anoxybacillus*
**FB4_71**	Yes	+/+/+	Bacteria; Firmicutes; Bacilli; Bacillales; Bacillaceae; *Anoxybacillus*
FB4_72	Yes	+/–/+	Bacteria; Firmicutes; Bacilli; Bacillales; Bacillaceae; *Anoxybacillus*
FB4_73	Yes	+/–/–	Bacteria; Firmicutes; Bacilli; Bacillales; Bacillaceae; *Anoxybacillus*
FB4_74	Yes	–/–/+	Bacteria; Firmicutes; Bacilli; Bacillales; Bacillaceae; *Brevibacillus*
**FB4_75**	Yes	+/–/+	Bacteria; Firmicutes; Bacilli; Bacillales; Bacillaceae; *Brevibacillus*
FB4_76	Yes	–/–/–	Bacteria; Firmicutes; Bacilli; Bacillales; Bacillaceae; *Brevibacillus*
**FB4_77**	Yes	+/+/+	Bacteria; Firmicutes; Bacilli; Bacillales; Bacillaceae; *Brevibacillus*
**FB4_79**	Yes	++/++/+++	Bacteria; Firmicutes; Bacilli; Bacillales; Bacillaceae; *Geobacillus*
**FB4_80**	Yes	+/–/+	Bacteria; Firmicutes; Bacilli; Bacillales; Bacillaceae; *Geobacillus*
FB4_81	No	n.t.	Bacteria; Firmicutes; Bacilli; Bacillales; Bacillaceae; *Geobacillus*
FB4_82	No	n.t.	Bacteria; Firmicutes; Bacilli; Bacillales; Bacillaceae; *Geobacillus*
FB4_84	Yes	+/–/–	Bacteria; Firmicutes; Bacilli; Bacillales; Bacillaceae; *Anoxybacillus*
**FB4_85**	Yes	+++/+++/+++	Bacteria; Firmicutes; Bacilli; Bacillales; Bacillaceae; *Geobacillus*
FB4_86	Yes	–/–/+	Bacteria; Firmicutes; Bacilli; Bacillales; Bacillaceae; *Geobacillus*
FB4_87	Yes	+/–/–	Bacteria; Firmicutes; Bacilli; Bacillales; Bacillaceae; *Geobacillus*
**FB4_88**	Yes	+++/+++/+++	Bacteria; Firmicutes; Bacilli; Bacillales; Bacillaceae; *Anoxybacillus*
**FB4_89**	Yes	–/+/+	Bacteria; Firmicutes; Bacilli; Bacillales; Bacillaceae; *Geobacillus*
FB4_90	Yes	–/–/+	Bacteria; Firmicutes; Bacilli; Bacillales; Bacillaceae; *Geobacillus*
**FB4_91**	Yes	–/+++/+++	Bacteria; Firmicutes; Bacilli; Bacillales; Bacillaceae; *Geobacillus*
**FB4_93**	Yes	++/++/+++	Bacteria; Firmicutes; Bacilli; Bacillales; Bacillaceae; *Geobacillus*
**FB4_94**	Yes	++/+/++	Bacteria; Firmicutes; Bacilli; Bacillales; Bacillaceae; *Geobacillus*
**FB4_95**	Yes	+/–/–	Bacteria; Firmicutes; Bacilli; Bacillales; Bacillaceae; *Geobacillus*
FB4_96	Yes	–/–/–	Bacteria; Firmicutes; Bacilli; Bacillales; Bacillaceae; *Geobacillus*
FB4_97	No	n.t.	Bacteria; Firmicutes; Bacilli; Bacillales; Bacillaceae; *Geobacillus*
FB4_99	Yes	+/–/–	Bacteria; Firmicutes; Bacilli; Bacillales; Bacillaceae; *Geobacillus*
**FB4_100**	Yes	+/–/+	Bacteria; Firmicutes; Bacilli; Bacillales; Bacillaceae; *Geobacillus*
**FB4_101**	Yes	+/–/+	Bacteria; Firmicutes; Bacilli; Bacillales; Bacillaceae; *Geobacillus*
FB4_102	Yes	+/–/–	Bacteria; Firmicutes; Bacilli; Bacillales; Bacillaceae; *Geobacillus*
**FB4_103**	Yes	+/+/++	Bacteria; Firmicutes; Bacilli; Bacillales; Bacillaceae; *Geobacillus*
FB4_104	Yes	+/–/–	Bacteria; Firmicutes; Bacilli; Bacillales; Bacillaceae; *Geobacillus*
**FB4_105**	Yes	+/–/–	Bacteria; Firmicutes; Bacilli; Bacillales; Bacillaceae; *Geobacillus*
FB4_109	No	n.t.	Bacteria; Firmicutes; Bacilli; Bacillales; Bacillaceae; *Anoxybacillus*
FB4_118	No	n.t.	Bacteria; Firmicutes; Bacilli; Bacillales; Bacillaceae; *Geobacillus*
**WB1_122**	Yes	++/++/+++	Bacteria; Firmicutes; Bacilli; Bacillales; Bacillaceae; *Geobacillus*
WB1_123	Yes	–/–/–	Bacteria; Firmicutes; Bacilli; Bacillales; Bacillaceae; *Anoxybacillus*
WB1_124	Yes	+/–/–	Bacteria; Firmicutes; Bacilli; Bacillales; Bacillaceae; *Anoxybacillus*
**WB1_125**	Yes	++/+/++	Bacteria; Firmicutes; Bacilli; Bacillales; Bacillaceae; *Anoxybacillus*
WB1_126	Yes	+/–/–	Bacteria; Firmicutes; Bacilli; Bacillales; Bacillaceae; *Anoxybacillus*
WB1_127	Yes	+/–/–	Bacteria; Firmicutes; Bacilli; Bacillales; Bacillaceae; *Anoxybacillus*
**WB1_128**	Yes	+/+/+	Bacteria; Firmicutes; Bacilli; Bacillales; Bacillaceae; *Anoxybacillus*
WB1_133	Yes	+/–/–	Bacteria; Firmicutes; Bacilli; Bacillales; Bacillaceae; *Brevibacillus*
**WB1_134**	Yes	+/–/+	Bacteria; Firmicutes; Bacilli; Bacillales; Bacillaceae; *Brevibacillus*
**WB1_135**	Yes	+/+/+	Bacteria; Firmicutes; Bacilli; Bacillales; Bacillaceae; *Brevibacillus*
**WB1_136**	Yes	+/+/++	Bacteria; Firmicutes; Bacilli; Bacillales; Bacillaceae; *Brevibacillus*
**WB1_137**	Yes	+/–/+	Bacteria; Firmicutes; Bacilli; Bacillales; Bacillaceae; *Brevibacillus*
WB1_138	No	n.t.	Bacteria; Firmicutes; Bacilli; Bacillales; Bacillaceae; *Geobacillus*
WB2_144	Yes	–/–/–	Bacteria; Firmicutes; Bacilli; Bacillales; Bacillaceae; *Brevibacillus*
**WB2_145**	Yes	+/–/–	Bacteria; Firmicutes; Bacilli; Bacillales; Bacillaceae; *Brevibacillus*
**WB2_150**	Yes	+/–/+	Bacteria; Firmicutes; Bacilli; Bacillales; Bacillaceae; *Brevibacillus*
WB2_152	No	n.t.	Bacteria; Firmicutes; Bacilli; Bacillales; Bacillaceae; *Geobacillus*
**WB2_153**	Yes	+/+/++	Bacteria; Firmicutes; Bacilli; Bacillales; Bacillaceae; *Geobacillus*
WB2_159	No	n.t.	Bacteria; Firmicutes; Bacilli; Bacillales; Bacillaceae; *Anoxybacillus*

*n.t., not tested; –, negative result for oil degradation; +, weak;++, strong; +++, very strong.*

*Strains in bold are those selected for biosurfactant production screening.*

For the screening of biosurfactant and bioemulsifier production, 50 isolates capable of degrading oil were tested by different methods (Table 2). Emulsification tests were performed using crude oil and diesel. More than half of the thermophilic isolates were able to emulsify at least one of the hydrocarbons used (*n* = 32). Most of the emulsion values (%) of the strains using crude oil were higher than those obtained with diesel, except for strain FB4_89 (*Geobacillus*), which showed better results for diesel (EI = 56% in diesel and 35% in crude oil). In total, 24 of the 50 thermophilic bacterial strains showed IE% higher than 30%; 21 were from Fumarole Bay and 3 from Whalers Bay. The great majority of strains with EI% higher than 30% belonged to *Geobacillus* and were from Fumarole Bay, with 12 representatives (two from Whalers Bay), followed by *Anoxybacillus*, with seven isolates (one from Whalers Bay).

**TABLE 2:** Strains isolated from different geothermal sites in Deception Island, their capacity to produce biosurfactants using different methodologies for screening (all in triplicate) and their molecular identification.

Isolate code	E24–Crude oil EI (%)	E24–Diesel EI (%)	E24–Crude oil EI (%)–100°C	E24–Diesel EI (%)–100°C	Drop-collapse test	Oil spreading assay (cm)	Hemolysis test	SILVA v. 132 classification
FB1_1	35	15	11	15	–/–/–	1.6 +/– 0.14	+/+/+	Bacteria; Firmicutes; Bacilli; Bacillales; Bacillaceae; *Anoxybacillus*
FB1_3	–	–	n.t.	n.t.	–/–/–	–	–/–/–	Bacteria; Firmicutes; Bacilli; Bacillales; Bacillaceae; *Anoxybacillus*
FB1_12	–	–	n.t.	n.t.	–/–/–	–	–/–/–	Bacteria; Firmicutes; Bacilli; Bacillales; Bacillaceae; *Geobacillus*
FB1_13	–	–	n.t.	n.t.	–/–/–	–	–/–/–	Bacteria; Firmicutes; Bacilli; Bacillales; Bacillaceae; *Geobacillus*
FB1_14	35	4	35	12	+/+/+	1.9 +/– 0.06	+/+/+	Bacteria; Firmicutes; Bacilli; Bacillales; Bacillaceae; *Geobacillus*
FB2_25	30	5	20	15	+/+/+	1.1 +/– 0.05	+/+/+	Bacteria; Firmicutes; Bacilli; Bacillales; Bacillaceae; *Anoxybacillus*
FB2_26	–	10	n.t.	n.t.	–/–/–	1.2 +/– 0.05	–/–/–	Bacteria; Firmicutes; Bacilli; Bacillales; Bacillaceae; *Anoxybacillus*
FB2_27	–	–	n.t.	n.t.	–/–/–	1.4 +/– 0.03	–/–/–	Bacteria; Firmicutes; Bacilli; Bacillales; Bacillaceae; *Anoxybacillus*
FB2_29	–	–	n.t.	n.t.	–/–/–	–	–/–/–	Bacteria; Firmicutes; Bacilli; Bacillales; Bacillaceae; *Anoxybacillus*
FB2_31	–	–	n.t.	n.t.	–/–/–	1.7 +/– 0.05	–/–/–	Bacteria; Firmicutes; Bacilli; Bacillales; Bacillaceae; *Anoxybacillus*
FB2_35	30	6	20	30	+/+/+	2.3 +/– 0.06	–/–/–	Bacteria; Firmicutes; Bacilli; Bacillales; Bacillaceae; *Geobacillus*
**FB2_36**	50	12	25	20	+/+/+	4.4 +/– 0.18	+/+/+	Bacteria; Firmicutes; Bacilli; Bacillales; Bacillaceae; *Geobacillus*
**FB2_38**	50	40	50	40	+/+/+	4.2 +/– 0.14	–/–/–	Bacteria; Firmicutes; Bacilli; Bacillales; Bacillaceae; *Geobacillus*
FB3_44	20	–	n.t.	n.t.	+/+/+	2.0 +/– 0.05	–/–/–	Bacteria; Firmicutes; Bacilli; Bacillales; Bacillaceae; *Anoxybacillus*
FB3_47	–	–	n.t.	n.t.	–/–/–	–	–/–/–	Bacteria; Firmicutes; Bacilli; Bacillales; Bacillaceae; *Geobacillus*
**FB3_49**	50	30	50	30	+/+/+	3.6 +/– 0.03	+/+/+	Bacteria; Firmicutes; Bacilli; Bacillales; Bacillaceae; *Geobacillus*
FB3_50	–	–	n.t.	n.t.	–/–/–	–	–/–/–	Bacteria; Firmicutes; Bacilli; Bacillales; Bacillaceae; *Geobacillus*
**FB3_54**	50	4	50	6	–/–/–	3.8 +/– 0.14	+/+/+	Bacteria; Firmicutes; Bacilli; Bacillales; Bacillaceae; *Geobacillus*
FB3_58	10	10	n.t.	n.t.	–/–/–	–	–/–/–	Bacteria; Firmicutes; Bacilli; Bacillales; Bacillaceae; *Geobacillus*
FB4_66	45	10	45	15	–/–/–	1.2 +/– 0.05	+/+/+	Bacteria; Firmicutes; Bacilli; Bacillales; Bacillaceae; *Brevibacillus*
**FB4_67**	50	4	50	10	–/–/–	4.2 +/– 0.13	+/+/+	Bacteria; Firmicutes; Bacilli; Bacillales; Bacillaceae; *Anoxybacillus*
**FB4_68**	50	8	45	10	–/–/–	4.0 +/– 0.01	+/+/+	Bacteria; Firmicutes; Bacilli; Bacillales; Bacillaceae; *Anoxybacillus*
FB4_69	35	11	30	15	–/–/–	–	+/+/+	Bacteria; Firmicutes; Bacilli; Bacillales; Bacillaceae; *Anoxybacillus*
FB4_70	–	–	n.t.	n.t.	–/–/–	–	–/–/–	Bacteria; Firmicutes; Bacilli; Bacillales; Bacillaceae; *Anoxybacillus*
FB4_71	–	–	n.t.	n.t.	–/–/–	–	–/–/–	Bacteria; Firmicutes; Bacilli; Bacillales; Bacillaceae; *Anoxybacillus*
FB4_75	–	10	n.t.	n.t.	–/–/–	–	–/–/–	Bacteria; Firmicutes; Bacilli; Bacillales; Bacillaceae; *Brevibacillus*
FB4_77	40	25	40	25	+/+/+	2.4 +/– 0.11	–/–/–	Bacteria; Firmicutes; Bacilli; Bacillales; Bacillaceae; *Brevibacillus*
FB4_79	–	–	n.t.	n.t.	–/–/–	–	–/–/–	Bacteria; Firmicutes; Bacilli; Bacillales; Bacillaceae; *Geobacillus*
FB4_80	–	–	n.t.	n.t.	–/–/–	–	–/–/–	Bacteria; Firmicutes; Bacilli; Bacillales; Bacillaceae; *Geobacillus*
FB4_85	35	20	40	20	+/+/+	1.7 +/– 0.06	+/+/+	Bacteria; Firmicutes; Bacilli; Bacillales; Bacillaceae; *Geobacillus*
**FB4_88**	50	40	50	40	+/+/+	5.7 +/– 0.25	+/+/+	Bacteria; Firmicutes; Bacilli; Bacillales; Bacillaceae; *Anoxybacillus*
**FB4_89**	35	56	100	60	+/+/+	5.0 +/– 0.02	+/+/+	Bacteria; Firmicutes; Bacilli; Bacillales; Bacillaceae; *Geobacillus*
**FB4_91**	50	35	50	35	+/+/+	5.1 +/– 0.03	+/+/+	Bacteria; Firmicutes; Bacilli; Bacillales; Bacillaceae; *Geobacillus*
FB4_93	40	8	50	14	+/+/+	3.3 +/– 0.17	+/+/+	Bacteria; Firmicutes; Bacilli; Bacillales; Bacillaceae; *Geobacillus*
FB4_94	–	–	n.t.	n.t.	–/–/–	–	–/–/–	Bacteria; Firmicutes; Bacilli; Bacillales; Bacillaceae; *Geobacillus*
FB4_95	–	–	n.t.	n.t.	–/–/–	–	–/–/–	Bacteria; Firmicutes; Bacilli; Bacillales; Bacillaceae; *Geobacillus*
FB4_100	15	–	n.t.	n.t.	+/+/+	2.4 +/– 0.05	–/–/–	Bacteria; Firmicutes; Bacilli; Bacillales; Bacillaceae; *Anoxybacillus*
**FB4_101**	50	20	50	20	–/–/–	4.3 +/– 0.29	+/+/+	Bacteria; Firmicutes; Bacilli; Bacillales; Bacillaceae; *Geobacillus*
FB4_103	10	–	n.t.	n.t.	+/+/+	2.7 +/– 0.17	–/–/–	Bacteria; Firmicutes; Bacilli; Bacillales; Bacillaceae; *Geobacillus*
**FB4_105**	55	8	100	10	+/+/+	4.0 +/– 0.11	+/+/+	Bacteria; Firmicutes; Bacilli; Bacillales; Bacillaceae; *Geobacillus*
**WB1_122**	60	6	55	8	–/–/–	3.5 +/– 0.02	+/+/+	Bacteria; Firmicutes; Bacilli; Bacillales; Bacillaceae; *Geobacillus*
WB1_125	–	–	n.t.	n.t.	+/+/+	3.0 +/– 0.05	–/–/–	Bacteria; Firmicutes; Bacilli; Bacillales; Bacillaceae; *Anoxybacillus*
WB1_128	40	8	40	23	–/–/–	2.4 +/– 0.19	–/–/–	Bacteria; Firmicutes; Bacilli; Bacillales; Bacillaceae; *Anoxybacillus*
WB1_134	–	10	n.t.	n.t.	–/–/–	–	–/–/–	Bacteria; Firmicutes; Bacilli; Bacillales; Bacillaceae; *Brevibacillus*
WB1_135	–	–	n.t.	n.t.	–/–/–	–	–/–/–	Bacteria; Firmicutes; Bacilli; Bacillales; Bacillaceae; *Brevibacillus*
WB1_136	10	5	n.t.	n.t.	–/–/–	–	+/+/+	Bacteria; Firmicutes; Bacilli; Bacillales; Bacillaceae; *Brevibacillus*
WB1_137	–	–	n.t.	n.t.	–/–/–	–	–/–/–	Bacteria; Firmicutes; Bacilli; Bacillales; Bacillaceae; *Brevibacillus*
WB2_145	10	6	n.t.	n.t.	–/–/–	–	–/–/–	Bacteria; Firmicutes; Bacilli; Bacillales; Bacillaceae; *Brevibacillus*
WB2_150	–	–	n.t.	n.t.	–/–/–	–	–/–/–	Bacteria; Firmicutes; Bacilli; Bacillales; Bacillaceae; *Brevibacillus*
**WB2_153**	55	30	60	30	+/+/+	3.7 +/– 0.14	+/+/+	Bacteria; Firmicutes; Bacilli; Bacillales; Bacillaceae; *Geobacillus*

*–, negative result for emulsification; +, positive result; -, negative result; n.t., not tested (EI ≤ 30%).*

*Strains in bold are those with the better results in the screening for biosurfactant production (EI ≥ 50% for one petroleum hydrocarbon source).*

Biosurfactant- and bioemulsifier-producing isolates that showed emulsification higher than 30% in the E24 assay were also tested for emulsification stability at high temperatures after treated at 100°C. For this analysis, 24 samples were tested in tubes containing crude oil and diesel. Most of the emulsion values (%) of strains using crude oil were higher than those obtained with diesel after treated at 100°C. Biosurfactant stability in crude oil was higher in strains isolated from sediments from geothermal environments with higher temperatures (FB3 and FB4), with lower emulsification indexes in strains isolated from the cooler environments. Values for the biosurfactant stability in diesel after treated at 100°C were similar to or higher than those found at room temperature, indicating higher emulsifying activity at high temperatures; however, the values were not higher than observed for crude oil. Strains FB2_38, FB4_91, FB4_88, FB4_89, and WB2_153 showed promising results for crude oil and diesel emulsification when treated at 100°C and non-treated (Table 2).

All strains that did not show emulsification activity also showed negative results in the drop-collapse, oil spreading, and hemolytic activity tests. Of the 50 thermophilic strains analyzed, 58% were positive for the oil-spreading assay and 36% positive for the drop-collapse test. For hemolytic activity, 40% of the isolates showed positive results in blood-agar plates; most were members of *Geobacillus* (Table 2).

In total, 13 thermophilic isolates showed particularly good results in the search for biosurfactant producers, with IEs of 50% or more in one of the petroleum hydrocarbons sources, positive results in the other tests and for high-temperature emulsification stability. Ten of these strains were from Fumarole Bay: FB2_36, FB2_38, FB2_49, FB3_54, FB4_67, FB4_68, FB4_88, FB4_91, FB4_101, and FB4_105, and three from Whalers Bay: WB1_122, WB1_128, and WB2_153. Of these, 4 isolates also showed great ability to degrade crude oil: FB2_38 (*Geobacillus*), FB3_54 (*Geobacillus*), FB4_88 (*Anoxybacillus*), and WB1_122 (*Geobacillus*). Additionally, it is worth to mention that the strains from Fumarole Bay were also able of growing under hyperthermophilic conditions (100°C).

### Genomic Analysis of an Oil-Degrading and Biosurfactant Producer Bacterium

The genome of the strain identified as *Anoxybacillus* FB4_88 was assembled *de novo*. The genome size was 2.9 Mb, with 41.9% GC content and 3,160 protein coding sequences in 209 contigs. General features of *Anoxybacillus* FB4_88 and strains of Bacillaceae are summarized in Table 3.

**TABLE 3:** General features of the genome of *Anoxybacillus* FB4_88 and genomes of other members of Bacillaceae.

Strain	Genome size (bp)	GC content (%)	Number of CDS	Number of RNA	Assembly and annotation level	Isolate origin	Accession number
FB4_88	2,901,684	41.9	3,160	64	209 contigs	Volcanic sediment, Antarctica	JAKNCX000000000
*Anoxybacillus flavithermus* WK1	2,846,746	41.8	2,863	104	Complete genome	Geothermal power station, New Zealand	GCA_000019045.1
*Anoxybacillus flavithermus* TNO-09.006	2,658,425	42.0	2,761	53 tRNA	1 scaffold	Dairy-production plants, Netherlands	GCA_000327465.1
*Anoxybacillus flavithermus* NBRC 109594	2,772,624	41.6	2,883	80 tRNA	90 contigs	Hot spring, Japan	GCA_000367505.1
*Anoxybacillus amylolyticus* DSM 15939T	3,158,269	43.5	3,092	108	Complete genome	Volcanic sediment, Antarctica	GCA_001634285.1
*Anoxybacillus* sp. B7M1	3,814,080	n.a	3,558	130	Complete genome	Geothermal environment, Germany	GCA_001634305.1
*Geobacillus* sp. 12AMOR1	3,410,035	52.0	3,323	122	Complete genome	Deep-sea hydrothermal vent, Arctic	GCA_001028085.1

*n.a, not available.*

**FIGURE 4:**
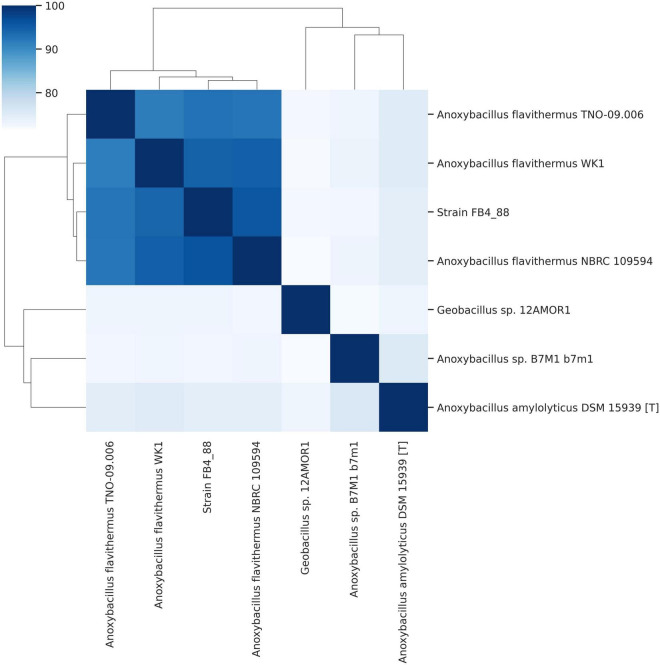
Genomic comparison between different strains of *Anoxybacillus* analyzed in this study and Bacillaceae members, using ANIb (in %). On the basis of the % ANIb, the analyzed strains formed two groups, and the analyzed genome clustered with *Anoxybacillus flavithermus* strains.

Based on 16S rRNA gene sequencing, the Antarctic strain was phylogenetically affiliated with *Anoxybacillus*. Applying phylogenomic analysis using the complete distance matrix genome (ANI) and Bacillaceae genomes from public databases, strain FB4_88 was phylogenomically closest to *Anoxybacillus flavithermus*, with 96% similarity for ANIb. Comparison of the Antarctic strain with other representatives of Bacillaceae (*A. amylolyticus, Anoxybacillus* sp., and *Geobacillus* sp.) resulted in ANIb values lower than 75% (Figure 4 and Supplementary Table 3).

Orthologous clusters were annotated with OrthoVenn2 comparing the groups of the strain FB4_88 and genomes of *Anoxybacillus* and *Geobacillus* analyzed. The species formed 4,342 clusters, of which 3,638 were orthologous (containing at least two species) and 704 were single-copy gene clusters. All the genomes shared 1,644 orthologous clusters; 113 orthologous clusters were shared between the *Anoxybacillus* strains, and 112 shared between the strains of *A. flavithermus*, indicating a high number of conserved (shared) genes among these genomes (Supplementary Figure 2).

**FIGURE 5:**
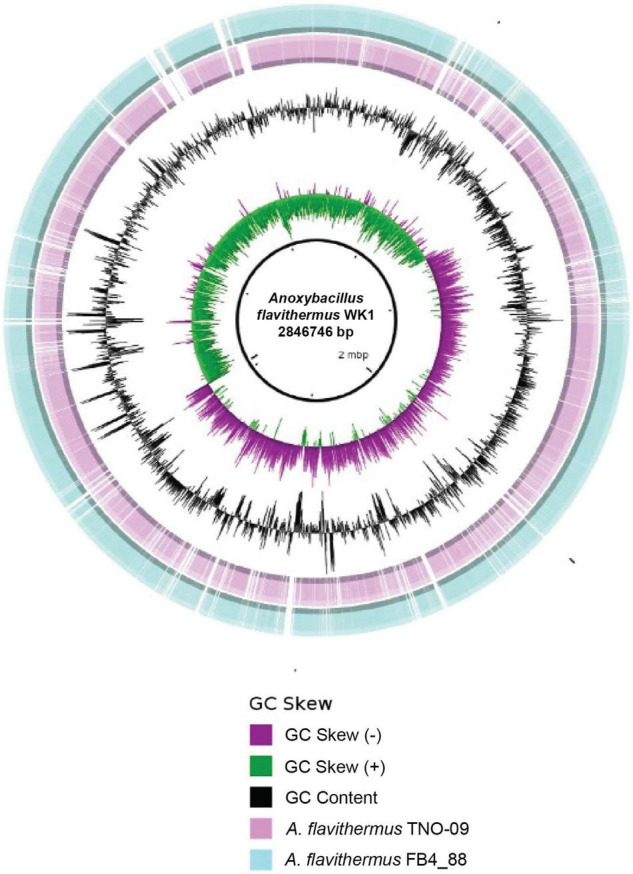
Genome comparison of *Anoxybacillus* species. BRIG image with the genome sequence of strain WK1 set as the central reference, while other genomes (strains TNO-09 and FB4_88 isolated in this study) were set as concentric rings. The inner black circle comprises the complete reference genome; the intensity of each color indicates the degree of similarity of that strain to the reference genome.

A circular visualization of the genome comparisons of the *Anoxybacillus* strains was created by BRIG (Figure 5). The inner black circle comprises the complete reference genome, corresponding to *A. flavithermus* WK1, with 2.8 Mb; the intensity of each color indicates the degree of similarity of that strain to the reference genome. The output image showed high similarity between FB4_88 and the other two genomes, mainly with WK1.

General functional annotations were performed with GOFEAT and RAST. Outputs from GOFEAT showed a sequence coverage of 63%; of these, more than 50% were related to molecular functions, followed by biological processes (25.7%) and cellular components (23.7%). RAST subsystems related to amino acids and their derivatives, carbohydrates, protein metabolism, and cofactors, vitamins, prosthetic groups, and pigments showed the highest numbers of assigned genes, in descending order.

Metabolism of aromatic compounds showed three subsystems in the RAST annotation - quinate degradation, salicylate and gentisate catabolism, and gentisate degradation. In agreement with these results and showing robust outcomes, pathways related to hydrocarbon biodegradation and biosurfactant production were identified through BiosurfDB. Additionally, BiosurfDB, a specialized platform for hydrocarbon degradation annotation, retrieved different coding-genes involved in this process.

**FIGURE 6:**
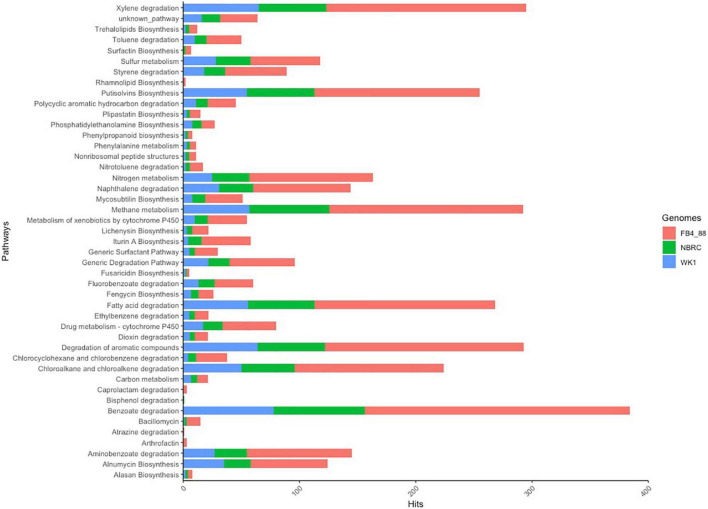
Functional annotation of *Anoxybacillus flavithermus* strains (NBRC, WK1, and FB4_88) for biodegradation of petroleum hydrocarbons and biosurfactant production. *A. flavithermus* FB4_88 isolated in this study is represented in blue.

All annotated pathways are shown in Figure 6. The highest numbers of hits were related to benzoate degradation, xylene degradation, and degradation of aromatic compounds, respectively. Remarkably, most of the pathways (32 of 45) showed significantly more hits in the strain isolated at Deception Island, compared with other *Anoxybacillus* strains. For the pathway for benzoate degradation, the most hits were retrieved for strain FB4_88 (*n* = 228), followed by strain WK1 (*n* = 78) and only two hits for strain NBRC. The same pattern was observed for xylene degradation, nitrogen metabolism, chloroalkane and chloroalkane degradation, naphthalene degradation, and cytochrome P450. Coding-genes such as *nah*C, *phe*A, and *bup*G were also identified and were highly abundant in the genome; they are related to hydrocarbon degradation, involved in the naphthalene catabolic pathway, toluene and xylene oxidation in the mono-oxygenase pathway, and alkylphenol degradation, respectively. Interestingly, the FB4_88 genetic system did not show *alk*B, an alkane hydroxylase responsible for aerobic alkane degradation and commonly found in oil-degrading bacteria.

Regarding the biosurfactant-production pathways, important members of the lipopeptide family were present, such as putisolvins (the most hits), Iturin A, lichenysin, fengycin, and bacillomycin biosynthesis; also surfactin and rhamnolipid biosynthesis were detected (with few hits) in strain FB4_88 and were not identified in the reference genome of strain WK1. Coding-genes for biosurfactant production were identified in the genomes. The gene *srf*A, which codes for surfactin synthase subunit 1, a protein involved in the surfactin biosynthesis pathway, and the *pps*A gene, involved in the gluconeogenesis pathway were predicted, as well as the gene *aln*B, part of the gene cluster that mediates biosynthesis of the polyketide asperlin.

Additionally, secondary metabolites were predicted with antiSMASH, PRISM, and BAGEL. The analyzed genome exhibited two gene clusters, one assigned as a non-ribosomal peptide synthetase cluster (NPRS)/betalactone, with 46% similarity with fengycin, as well as a cluster assigned to type III PKS (T3PKS). PRISM 4 detected one non-ribosomal peptide, while with BAGEL, sactipeptides - a member of the RiPP family, were identified in two areas of interest. Using PHASTER, only one incomplete prophage sequence was detected in the FB4_88 genome, related to *Bacillus*, with a GC content of 36.39%.

## Discussion

This study is the first attempt to assess culturable thermophilic bacteria from the Deception Island volcano, with the capacity to degrade petroleum hydrocarbon and produce biosurfactants and bioemulsifiers under thermophilic conditions. We isolated over 120 strains from two geothermal sites, using different culture media. The phylogenetic analysis classified all the isolates as belonging to Firmicutes. Members of Firmicutes have different morphological and physiological characteristics; the formation of heat-resistant endospores is a specific property of these bacteria, which makes them able to inhabit extreme environments, such as Antarctic volcanoes. Partial sequencing of the 16S rRNA gene revealed that the 126 cultured thermophilic isolates belonged to *Geobacillus, Brevibacillus*, and *Anoxybacillus*. These groups were reported previously in sediments associated with fumaroles from Deception Island (Llarch et al., 1997; Muñoz et al., 2011), as well as in glaciers at these locations (Bendia et al., 2018a). Several species of *Bacillus, Alicyclobacillus, Brevibacillus, Aneurinibacillus*, and *Anoxybacillus* have been isolated from other geothermal environments in Antarctica (Bargagli et al., 2004; Poli et al., 2006).

We tested the isolates for their potential ability to degrade crude oil to use as a carbon source; over 50% of the isolates were able to grow in oil under thermophilic conditions. Hydrocarbon-degrading bacteria are commonly assigned to *Acinetobacter, Rhodococcus, Alcanivorax, Bacillus*, and *Pseudomonas*, which are among the best-known genera (Sharma et al., 2019; Sharma and Pandey, 2021). Although thermophiles have been widely described, only a few thermophilic hydrocarbon-degrading species have been reported (Das and Chandran, 2011; Ivanova et al., 2014). Firmicutes also contains petroleum hydrocarbon-degrading bacteria and has been observed in abundance in oil-contaminated environments (Beazley et al., 2012; Engel et al., 2017; Datta et al., 2018, 2020). Species found in the Deception sediments are also well known to be capable of degrading petroleum hydrocarbons. *Geobacillus* is widely distributed in different environments, especially at high temperatures, such as in geothermal environments and oil reservoirs. Several species of *Geobacillus* have recently been described as degraders of different hydrocarbons and may use a wide variety of hydrocarbons, especially crude oil, kerosene, and phenanthrene (Marchant and Banat, 2010). Strains of *Brevibacillus* and *Anoxybacillus* have also been identified as hydrocarbon degraders under thermophilic conditions (Xia et al., 2015), such as *Brevibacillus* sp. PDM-3, which degraded phenanthrene as a carbon source (Reddy et al., 2010).

We also screened the potential ability of thermophilic bacterial isolates to produce biosurfactants. These amphipathic molecules from extreme environments are increasingly being investigated for potential applications in bioremediation and MEOR processes, as they are sustainable alternatives to chemical surfactants and exhibit activity and stability under extreme conditions (Darvishi et al., 2011; Akbari et al., 2018). The results showed that 13 of the 50 strains tested were potential biosurfactant-producing strains, showing good emulsification (IE > 50%) of crude oil and diesel. Good emulsification is essential for biosurfactants to be considered promising for environmental and industrial applications (Banat et al., 2000). Other properties of biosurfactants, such as oil drop-collapse, oil spreading, and hemolytic activity, were also tested. As previously observed by Couto et al. (2015), the results of these tests were not closely related, i.e., strains that showed emulsification ability did not show significant results in oil spreading or hemolytic activity.

Deception Island and its extremophiles have been little studied regarding their biotechnological potential. There are few but interesting reports, such as that of a biosurfactant-producing strain identified as Bacillus licheniformis AL 1.1, collected in Kroner Lake, Whalers Bay (Deception Island, Antarctica). This Antarctic strain was able to produce the biosurfactant lichenisin, a lipopeptide, using different carbohydrate sources under mesophilic conditions (Coronel-León et al., 2015). Biosurfactant production was found in our studied strains of all genera, predominantly in *Geobacillus*. Studies describing biosurfactant production by *Geobacillus* species are very recent; until 2012, no studies were reported (Marchant and Banat, 2012). Xia et al. (2016) isolated a new strain of *Geobacillus* that showed biosurfactant production and high emulsification rates in different petroleum hydrocarbons (e.g., crude oil, kerosene, and hexane). Al-Jailawi et al. (2015) reported that *G. thermoleovorans* Ir1 showed a maximum crude oil emulsification of 68%. Jara et al. (2013) found that *G. stearothermophilus* isolated from an oil-contaminated soil produced biosurfactants with 87% emulsifying activity for oil. The present results agree with previous reports that members of *Geobacillus* have high potential for bioremediation of oil-contaminated environments and MEOR.

Isolates belonging to *Anoxybacillus* and *Brevibacillus* have also been reported as having a high emulsification capacity. According to Pakpitcharoena et al. (2008), the production of biosurfactants by thermophilic isolates of *Anoxybacillus* remain very scarce, with very few studies reporting them as biosurfactant producers. One of the studies reported that *Anoxybacillus* sp. WJ-4 produced an oligosaccharide–lipid–peptide bioemulsifier with a high emulsification index of 60% (Xia et al., 2015). A strain of *Brevibacillus brevis* isolated from an oil reservoir produced a lipopeptide-classified biosurfactant with an 80% emulsification index (Mouafi et al., 2016). Emulsification remained stable after treated with high temperatures. This feature is widely observed in biosurfactants, which are generally more effective in conditions of high salinity, pH, and temperature compared to synthetic surfactants (Cameotra et al., 2010). High temperature is a key parameter affecting emulsifying activity and bacterial growth, desired features for MEOR and oil reservoirs are high-temperature environments (Uad et al., 2010).

The genomic analysis confirmed the potential of the selected strain for hydrocarbon degradation and biosurfactant production, as screened in the bench analysis. This oil-degrading and biosurfactant-producing strain was identified as *Anoxybacillus flavithermus*, which occurs widely in thermophilic environments (Goh et al., 2014). Remarkably, compared with other strains of the genus, the Antarctic isolate showed a significantly higher number of hits for coding-genes and pathways related to hydrocarbon degradation, related mainly to benzoate, xylene, and naphthalene degradation. The gene *alk*B was not found in this genome, in agreement with Nie et al. (2014), who failed to find this gene in 1,077 genomes of Firmicutes sequenced. As mentioned above, genera of Firmicutes are well known as good candidates for oil-industry applications. However, only a few studies have assessed the potential of members of *Anoxybacillus* for use in this field. For example, *A. geothermalis* is able to degrade crude oil while producing alkane hydroxylase and lipase (Yusoff et al., 2020); and *Anoxybacillus* sp. WJ-4, a new thermophilic strain isolated in the Daqing oilfield, is capable of utilizing alkanes (C8–C22) as a sole source of carbon for growth and produced an oligosaccharide–lipid–peptide bioemulsifier with an EI% over 60%, with increased cell-surface lipophilicity to 65% during hydrocarbon degradation (Xia et al., 2015). Aside from culture studies in thermophilic environments, these habitats and their microbial communities in polar regions remain uninvestigated. Surprisingly, this is the first study to assess the genetic and functional capabilities of an Antarctic strain of *Anoxybacillus*.

As observed here for the potential degradation of crude oil, biosurfactant production, and high-temperature emulsification stability by members of Firmicutes, this phylum is not commonly found in substantial proportions in Antarctic environments, based on metagenomics data (Teixeira et al., 2010; Ramos et al., 2019). Recovery of the 16S gene from metagenomic samples from Deception Island gave the same result. Proteobacteria and Bacteroidetes dominated, in agreement with Bendia et al. (2018b), who analyzed the microbial community through 16S rRNA sequencing, and also observed the predominance of these phyla. Through these sequencing techniques, it is possible to sequence all the DNA present in a sample and obtain information from the microorganisms present. However, it is difficult to obtain the genomes of microorganisms that are relatively rare, and usually information is obtained only from the dominant populations (Albertsen et al., 2013; Jousset et al., 2017).

Our data suggest that Firmicutes, based on the under-representation in culture-independent analyses, is not the dominant phylum in Deception Island sediments, but may become dominant under optimal growing conditions or under some environmental interference (e.g., oil contamination). In this study, we attempted to culture the widest possible diversity of bacteria from growth media. However, even using several media in thermophilic conditions, we recovered only representatives of Firmicutes. Factors that might have led to this are the culture media used; the incubation time of up to 72 h, which could have led to isolation of fastidious microbes; and the logistics involved in transporting samples from Antarctica to the laboratory. For new culturomics studies, we strongly recommend using alternative culture approaches or designing specific isolation strategies to hamper microbial growth, such as the sprinkling method for microbial isolation instead of serial dilution; also, reducing nutrient availability and temperature and increasing the incubation time, as well as adding protective agents against oxidative stress and culturing in a consortium (Rocha, 2019), attempting to improve the culturability of other taxa from Deception sediments. Further studies are required to estimate the diversity of culturable microorganisms from geothermal environments of Antarctica and to exploit their metabolic potential for biotechnological applications.

In conclusion, through a variety of culture techniques, thermophilic isolates were obtained from two geothermal environments on Deception Island at temperatures ranging from 50 to 100°C, and identified as belonging to *Geobacillus, Anoxybacillus*, and *Brevibacillus*, which are spore-forming genera, suggesting the importance of spore formation for local resistance. Thermophiles capable of degrading petroleum and producing biosurfactants have been described for the first time from Deception Island, an active Antarctic volcano. Four strains gave the most promising results: FB2_38 (*Geobacillus*), FB3_54 (*Geobacillus*), FB4_88 (*Anoxybacillus*), and WB1_122 (*Geobacillus*). Additionally, genomic analysis was performed on strain FB4_88, further identified in this study as *Anoxybacillus flavithermus.* To the best of our knowledge, this is the first genome report on *A. flavithermus* isolated from volcanic sediment on Deception Island, and this strain exhibited a high genetic and functional diversity potential for biotechnology. Taken together, these initial insights from both culturomics and genomics data suggest that thermophilic bacteria from this Antarctic polar volcano are potential candidates for application in the petroleum industry, for possible bioremediation in extreme environments and for microbial enhanced oil recovery (MEOR) in oil reservoirs. In addition, non-dominant microorganisms may play an important role in hydrocarbon degradation and biosurfactant production in the Deception Island volcano sediments.

## Data Availability Statement

The datasets presented in this study can be found in online repositories. The names of the repository/repositories and accession number(s) can be found in the article/Supplementary Material.

## Author Contributions

JS and ASR: study conception and design. JS, ICVA, and RK: acquisition of data. JS, ICVA, UNR, and ASR: analyses and interpretation of data. JS, UNR, and ASR: drafting of the manuscript. All authors: critical revision. All authors contributed to the article and approved the submitted version.

## Conflict of Interest

The authors declare that the research was conducted in the absence of any commercial or financial relationships that could be construed as a potential conflict of interest.

## Publisher’s Note

All claims expressed in this article are solely those of the authors and do not necessarily represent those of their affiliated organizations, or those of the publisher, the editors and the reviewers. Any product that may be evaluated in this article, or claim that may be made by its manufacturer, is not guaranteed or endorsed by the publisher.

## Footnotes

1
www.geneious.com

2
https://www.uniprot.org/

3
https://www.ebi.ac.uk/interpro/

4
https://pfam.xfam.org/

5
https://www.ncbi.nlm.nih.gov/

6
https://www.genome.jp/kegg/
