# Supplementary material for: Polyphasic Analysis Reveals Potential Petroleum Hydrocarbon Degradation and Biosurfactant Production by Rare Biosphere Thermophilic Bacteria From Deception Island, an Active Antarctic Volcano
Source: Front Microbiol. 2022 May 4;13:885557. doi: 10.3389/fmicb.2022.885557 (PMC9114708; doi:10.3389/fmicb.2022.885557)
Supplement: Supplementary file 1 [file Data_Sheet_1.docx]

Supplementary Material

# Supplementary Figures

**
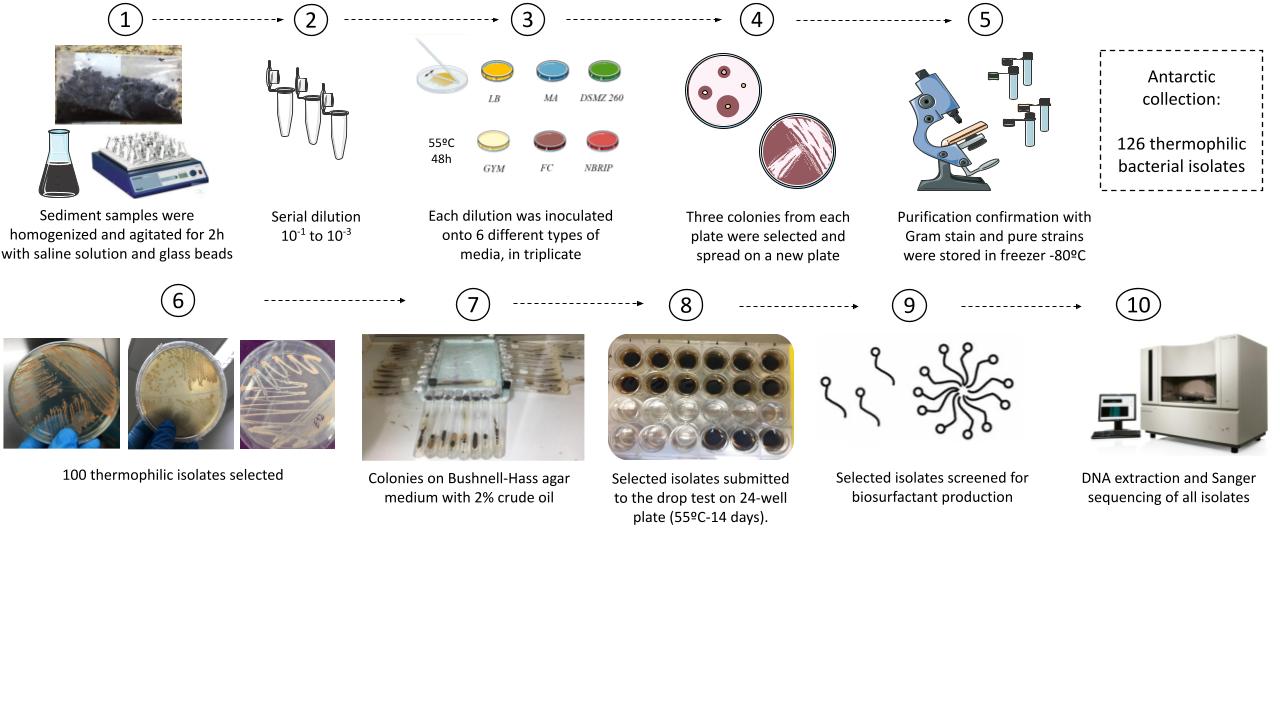
**

**Supplementary Figure 1** Scheme of culture steps (for description, see text).


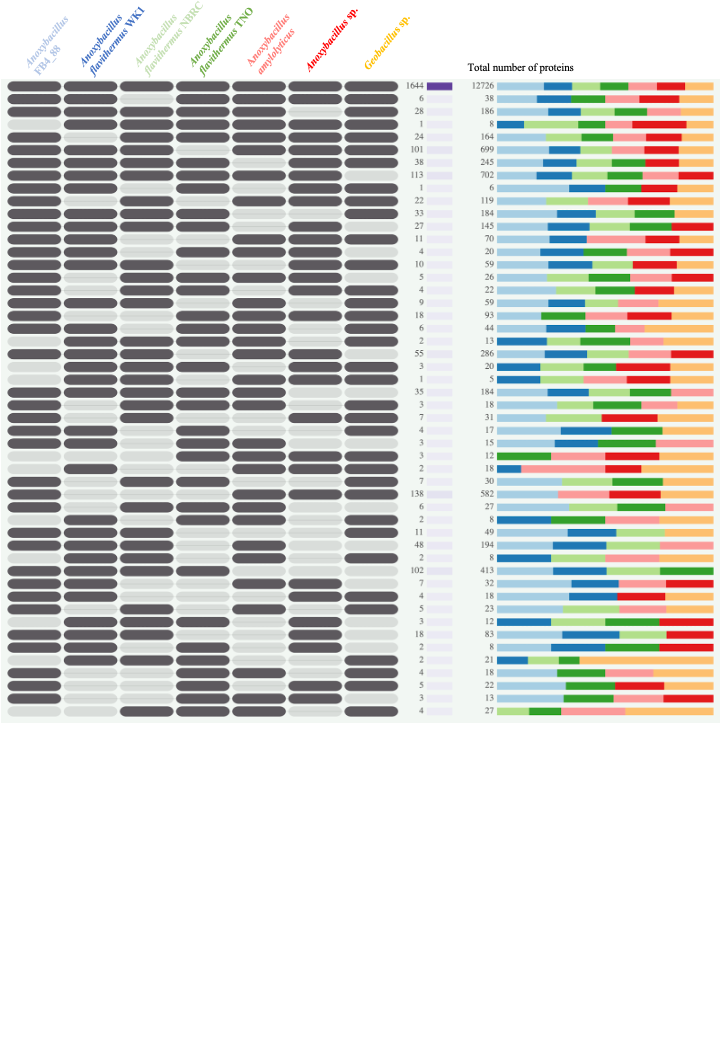


**Supplementary Figure 2** Orthologous groups belonging to the analyzed genome and the genomes of Bacillaceae closely annotated to our *Anoxybacillus* strain. From left to right: orthologous groups present in the genomes, in which the dark gray color indicates the presence of the orthologous groups in each strain while the light gray indicates their absence; in purple, number of protein orthologous groups shared in the genomes; and total number of proteins recorded in the shared orthologous groups, in which the colors represent the proportion of proteins belonging to each strain.

# Supplementary Tables

**Supplementary Table 1** Composition of the used culture media.

| **Culture media** | **Composition** | **Reference** |
| --- | --- | --- |
| Lysogeny agar | 10g Tryptone, 5g Yeast extract, 10g NaCl, 15g Agar, 1000ml Distilled water | Bertani, 1951 |
| Marine agar | 5g Peptone, 1g Yeast extract, 0.1g Ferric citrate, 19.450g Sodium chloride, 8.8g Magnesium chloride, 3.240g Sodium sulphate, 1.800g Calcium chloride, 0.550g Potassium chloride, 0.160g Sodium bicarbonate, 0.080g Potassium bromide, 0.034g Strontium chloride, 0.022g Boric acid, 0.004g Sodium silicate, 0.0024g Sodium fluorate, 0.0016g Ammonium nitrate, 0.008g Disodium phosphate, 15g Agar, 1000ml Distilled water | Zobell, 1941 |
| Glucose yeast malt medium | 4g Glucose, 4g Yeast extract, 10g Malt extract, 2g CaCO3, 12g Agar, 1000ml Distilled water | Mendez et al., 1985 |
| DSMZ 260 medium | 4.5g Na_2_HPO_4_ x 2 H_2_O, 1.5g KH_2_PO_4_, 1g NH_4_Cl, 0.01g MnSO_4_ x H_2_O, 0.2g MgSO_4_ x 7 H_2_O, 0.01g CaCl_2_ x 2 H_2_O, 5mg Ferric ammonium citrate, 3ml Trace element solution SL-6, 1.5g Na-Pyruvate, 15g agar, 1000ml Distilled water | DSMZ GmbH (https://www.dsmz.de/microorganisms/medium/pdf/DSMZ_Medium260.pdf) |
| Calcium phytate medium | 10g Glucose, 0.5g (NH_4_)_2_SO_4_, 0.2g KCl, 0.1g MgSO_4_ x 7 H_2_O, 2g Calcium phytate, 0.5g Yeast extract, 0.005g MnSO_4_, 0.005g FeSO_4_, 15g Agar, 1000ml Distilled water | Richardson et al., 2011 |
| National Botanical Research Institute Phosphate medium | 10g Glucose, 5g Calcium phosphate, 5g MgCL_2_ x 6 H_2_O, 0.25g MgSO_4_ x 7 H_2_O, 0.2g KCl  0.1g (NH_4_)_2_SO_4_, 0.025g Bromophenol blue, 15g Agar, 1000ml Distilled water | Nautiyal, 1999 |
| Lysogeny broth | 10g Tryptone, 5g Yeast extract, 10g NaCl, 1000ml Distilled water | Bertani, 1951 |
| Bushnell-Hass agar | 0.02g CaCl2 x 2 H2O, 0.2g MgSO_4_ x 7 H2O, 1g K_2_HPO_4_, 1g KH_2_PO_4_, 1g NH_4_NO_3_, 0.05g FeCl_3_ x 2 H_2_O, 15g Agar, 1000ml Distilled water | Atlas, 1995 |
| Bushnell-Hass broth | 0.02g CaCl2 x 2 H2O, 0.2g MgSO_4_ x 7 H2O, 1g K_2_HPO_4_, 1g KH_2_PO_4_, 1g NH_4_NO_3_, 0.05g FeCl_3_ x 2 H_2_O, 1000ml Distilled water | Atlas, 1995 |

**Supplementary Table 2** List of Deception thermophiles isolated from two geothermal sites, which were identified through 16S rRNA gene sequencing, including the environmental temperature of the sample (Celsius), percentage of sequence identity with database and their assigned taxonomy using SILVA v. 132 and BLASTn database, and the Genbank accession number.

| **Number** | **Isolate code** | **Local** | **Environmental temperature (ºC)** | **Identity (%)** | **SILVA v. 132 classification** | **NCBI taxonomy** | **Accession number (Genbank)** |
| --- | --- | --- | --- | --- | --- | --- | --- |
| 1 | FB1_1 | Fumarole Bay | 55ºC | 99 | Bacteria; Firmicutes; Bacilli; Bacillales; Bacillaceae; Anoxybacillus | *Anoxybacillus mongoliensis* | OM842843 |
| 2 | FB1_2 | Fumarole Bay | 55ºC | 99 | Bacteria; Firmicutes; Bacilli; Bacillales; Bacillaceae; Brevibacillus | *Brevibacillus thermoruber* | OM842844 |
| 3 | FB1_3 | Fumarole Bay | 55ºC | 99 | Bacteria; Firmicutes; Bacilli; Bacillales; Bacillaceae; Anoxybacillus | *Anoxybacillus mongoliensis* | OM842845 |
| 4 | FB1_4 | Fumarole Bay | 55ºC | 99 | Bacteria; Firmicutes; Bacilli; Bacillales; Bacillaceae; Brevibacillus | *Brevibacillus thermoruber* | OM842846 |
| 5 | FB1_5 | Fumarole Bay | 55ºC | 98 | Bacteria; Firmicutes; Bacilli; Bacillales; Bacillaceae; Brevibacillus | *Brevibacillus thermoruber* | OM842847 |
| 6 | FB1_6 | Fumarole Bay | 55ºC | 99 | Bacteria; Firmicutes; Bacilli; Bacillales; Bacillaceae; Brevibacillus | *Brevibacillus thermoruber* | OM842848 |
| 7 | FB1_7 | Fumarole Bay | 55ºC | 99 | Bacteria; Firmicutes; Bacilli; Bacillales; Bacillaceae; Brevibacillus | *Brevibacillus thermoruber* | OM842849 |
| 8 | FB1_8 | Fumarole Bay | 55ºC | 97 | Bacteria; Firmicutes; Bacilli; Bacillales; Bacillaceae; Geobacillus | *Geobacillus* sp. | OM842850 |
| 9 | FB1_9 | Fumarole Bay | 55ºC | 99 | Bacteria; Firmicutes; Bacilli; Bacillales; Bacillaceae; Geobacillus | *Geobacillus stearothermophilus* | OM842851 |
| 10 | FB1_10 | Fumarole Bay | 55ºC | 99 | Bacteria; Firmicutes; Bacilli; Bacillales; Bacillaceae; Geobacillus | *Geobacillus* sp. | OM842852 |
| 11 | FB1_11 | Fumarole Bay | 55ºC | 99 | Bacteria; Firmicutes; Bacilli; Bacillales; Bacillaceae; Geobacillus | *Geobacillus stearothermophilus* | OM842853 |
| 12 | FB1_12 | Fumarole Bay | 55ºC | 99 | Bacteria; Firmicutes; Bacilli; Bacillales; Bacillaceae; Geobacillus | *Geobacillus stearothermophilus* | OM842854 |
| 13 | FB1_13 | Fumarole Bay | 55ºC | 99 | Bacteria; Firmicutes; Bacilli; Bacillales; Bacillaceae; Geobacillus | *Geobacillus* sp. | OM842855 |
| 14 | FB1_14 | Fumarole Bay | 55ºC | 99 | Bacteria; Firmicutes; Bacilli; Bacillales; Bacillaceae; Geobacillus | *Geobacillus stearothermophilus* | OM842856 |
| 15 | FB1_15 | Fumarole Bay | 55ºC | 88 | Bacteria; Firmicutes; Bacilli; Bacillales; Bacillaceae; Geobacillus | *Geobacillus* sp. | OM842857 |
| 16 | FB2_21 | Fumarole Bay | 70ºC | 98 | Bacteria; Firmicutes; Bacilli; Bacillales; Bacillaceae; Anoxybacillus | *Anoxybacillus mongoliensis* | OM842858 |
| 17 | FB2_22 | Fumarole Bay | 70ºC | 98 | Bacteria; Firmicutes; Bacilli; Bacillales; Bacillaceae; Anoxybacillus | *Anoxybacillus mongoliensis* | OM842859 |
| 18 | FB2_23 | Fumarole Bay | 70ºC | 99 | Bacteria; Firmicutes; Bacilli; Bacillales; Bacillaceae; Anoxybacillus | *Anoxybacillus flavithermus* | OM842860 |
| 19 | FB2_25 | Fumarole Bay | 70ºC | 100 | Bacteria; Firmicutes; Bacilli; Bacillales; Bacillaceae; Anoxybacillus | *Anoxybacillus flavithermus* | OM842861 |
| 20 | FB2_26 | Fumarole Bay | 70ºC | 100 | Bacteria; Firmicutes; Bacilli; Bacillales; Bacillaceae; Anoxybacillus | *Anoxybacillus tunisiense* | OM842862 |
| 21 | FB2_27 | Fumarole Bay | 70ºC | 99 | Bacteria; Firmicutes; Bacilli; Bacillales; Bacillaceae; Anoxybacillus | *Anoxybacillus* sp. | OM842863 |
| 22 | FB2_28 | Fumarole Bay | 70ºC | 100 | Bacteria; Firmicutes; Bacilli; Bacillales; Bacillaceae; Anoxybacillus | *Anoxybacillus mongoliensis* | OM842864 |
| 23 | FB2_29 | Fumarole Bay | 70ºC | 100 | Bacteria; Firmicutes; Bacilli; Bacillales; Bacillaceae; Anoxybacillus | *Anoxybacillus mongoliensis* | OM842865 |
| 24 | FB2_30 | Fumarole Bay | 70ºC | 99 | Bacteria; Firmicutes; Bacilli; Bacillales; Bacillaceae; Brevibacillus | *Brevibacillus* sp. | OM842866 |
| 25 | FB2_31 | Fumarole Bay | 70ºC | 99 | Bacteria; Firmicutes; Bacilli; Bacillales; Bacillaceae; Anoxybacillus | *Anoxybacillus tunisiense* | OM842867 |
| 26 | FB2_32 | Fumarole Bay | 70ºC | 100 | Bacteria; Firmicutes; Bacilli; Bacillales; Bacillaceae; Brevibacillus | *Brevibacillus thermoruber* | OM842868 |
| 27 | FB2_33 | Fumarole Bay | 70ºC | 99 | Bacteria; Firmicutes; Bacilli; Bacillales; Bacillaceae; Brevibacillus | *Brevibacillus thermoruber* | OM842869 |
| 28 | FB2_34 | Fumarole Bay | 70ºC | 100 | Bacteria; Firmicutes; Bacilli; Bacillales; Bacillaceae; Geobacillus | *Geobacillus stearothermophilus* | OM842870 |
| 29 | FB2_35 | Fumarole Bay | 70ºC | 100 | Bacteria; Firmicutes; Bacilli; Bacillales; Bacillaceae; Geobacillus | *Geobacillus* sp. | OM842871 |
| 30 | FB2_36 | Fumarole Bay | 70ºC | 100 | Bacteria; Firmicutes; Bacilli; Bacillales; Bacillaceae; Geobacillus | *Geobacillus* sp. | OM842872 |
| 31 | FB2_37 | Fumarole Bay | 70ºC | 99 | Bacteria; Firmicutes; Bacilli; Bacillales; Bacillaceae; Geobacillus | *Geobacillus* sp. | OM842873 |
| 32 | FB2_38 | Fumarole Bay | 70ºC | 100 | Bacteria; Firmicutes; Bacilli; Bacillales; Bacillaceae; Geobacillus | *Geobacillus* sp. | OM842874 |
| 33 | FB2_39 | Fumarole Bay | 70ºC | 97 | Bacteria; Firmicutes; Bacilli; Bacillales; Bacillaceae; Brevibacillus | *Brevibacillus thermoruber* | OM842875 |
| 34 | FB3_43 | Fumarole Bay | 80ºC | 100 | Bacteria; Firmicutes; Bacilli; Bacillales; Bacillaceae; Anoxybacillus | *Anoxybacillus flavithermus* | OM842876 |
| 35 | FB3_44 | Fumarole Bay | 80ºC | 99 | Bacteria; Firmicutes; Bacilli; Bacillales; Bacillaceae; Anoxybacillus | *Anoxybacillus tunisiense* | OM842877 |
| 36 | FB3_45 | Fumarole Bay | 80ºC | 98 | Bacteria; Firmicutes; Bacilli; Bacillales; Bacillaceae; Anoxybacillus | *Anoxybacillus mongoliensis* | OM842878 |
| 37 | FB3_46 | Fumarole Bay | 80ºC | 100 | Bacteria; Firmicutes; Bacilli; Bacillales; Bacillaceae; Geobacillus | *Geobacillus stearothermophilus* | OM842879 |
| 38 | FB3_47 | Fumarole Bay | 80ºC | 100 | Bacteria; Firmicutes; Bacilli; Bacillales; Bacillaceae; Geobacillus | *Geobacillus* sp. | OM842880 |
| 39 | FB3_48 | Fumarole Bay | 80ºC | 100 | Bacteria; Firmicutes; Bacilli; Bacillales; Bacillaceae; Geobacillus | *Geobacillus* sp. | OM842881 |
| 40 | FB3_49 | Fumarole Bay | 80ºC | 100 | Bacteria; Firmicutes; Bacilli; Bacillales; Bacillaceae; Geobacillus | *Geobacillus* sp. | OM842882 |
| 41 | FB3_50 | Fumarole Bay | 80ºC | 100 | Bacteria; Firmicutes; Bacilli; Bacillales; Bacillaceae; Geobacillus | *Geobacillus thermoleovorans* | OM842883 |
| 42 | FB3_51 | Fumarole Bay | 80ºC | 100 | Bacteria; Firmicutes; Bacilli; Bacillales; Bacillaceae; Geobacillus | *Geobacillus thermoleovorans* | OM842884 |
| 43 | FB3_52 | Fumarole Bay | 80ºC | 99 | Bacteria; Firmicutes; Bacilli; Bacillales; Bacillaceae; Geobacillus | *Geobacillus* sp. | OM842885 |
| 44 | FB3_53 | Fumarole Bay | 80ºC | 99 | Bacteria; Firmicutes; Bacilli; Bacillales; Bacillaceae; Geobacillus | *Geobacillus thermoleovorans* | OM842886 |
| 45 | FB3_54 | Fumarole Bay | 80ºC | 100 | Bacteria; Firmicutes; Bacilli; Bacillales; Bacillaceae; Geobacillus | *Geobacillus caldoxylosilyticus* | OM842887 |
| 46 | FB3_55 | Fumarole Bay | 80ºC | 99 | Bacteria; Firmicutes; Bacilli; Bacillales; Bacillaceae; Geobacillus | *Geobacillus* sp. | OM842888 |
| 47 | FB3_56 | Fumarole Bay | 80ºC | 100 | Bacteria; Firmicutes; Bacilli; Bacillales; Bacillaceae; Geobacillus | *Geobacillus* sp. | OM842889 |
| 48 | FB3_57 | Fumarole Bay | 80ºC | 100 | Bacteria; Firmicutes; Bacilli; Bacillales; Bacillaceae; Geobacillus | *Geobacillus thermoleovorans* | OM842890 |
| 49 | FB3_58 | Fumarole Bay | 80ºC | 100 | Bacteria; Firmicutes; Bacilli; Bacillales; Bacillaceae; Geobacillus | *Geobacillus kaustophilus* | OM842891 |
| 50 | FB4_62 | Fumarole Bay | 100ºC | 100 | Bacteria; Firmicutes; Bacilli; Bacillales; Bacillaceae; Anoxybacillus | *Anoxybacillus mongoliensis* | OM842892 |
| 51 | FB4_63 | Fumarole Bay | 100ºC | 100 | Bacteria; Firmicutes; Bacilli; Bacillales; Bacillaceae; Anoxybacillus | *Anoxybacillus* sp. | OM842893 |
| 52 | FB4_64 | Fumarole Bay | 100ºC | 99 | Bacteria; Firmicutes; Bacilli; Bacillales; Bacillaceae; Anoxybacillus | *Anoxybacillus mongoliensis* | OM842894 |
| 53 | FB4_65 | Fumarole Bay | 100ºC | 100 | Bacteria; Firmicutes; Bacilli; Bacillales; Bacillaceae; Anoxybacillus | *Anoxybacillus mongoliensis* | OM842895 |
| 54 | FB4_66 | Fumarole Bay | 100ºC | 99 | Bacteria; Firmicutes; Bacilli; Bacillales; Bacillaceae; Brevibacillus | *Brevibacillus thermoruber* | OM842896 |
| 55 | FB4_67 | Fumarole Bay | 100ºC | 99 | Bacteria; Firmicutes; Bacilli; Bacillales; Bacillaceae; Anoxybacillus | *Anoxybacillus flavithermus* | OM842897 |
| 56 | FB4_68 | Fumarole Bay | 100ºC | 99 | Bacteria; Firmicutes; Bacilli; Bacillales; Bacillaceae; Anoxybacillus | *Anoxybacillus tunisiense* | OM842898 |
| 57 | FB4_69 | Fumarole Bay | 100ºC | 100 | Bacteria; Firmicutes; Bacilli; Bacillales; Bacillaceae; Anoxybacillus | *Anoxybacillus flavithermus* | OM842899 |
| 58 | FB4_70 | Fumarole Bay | 100ºC | 100 | Bacteria; Firmicutes; Bacilli; Bacillales; Bacillaceae; Anoxybacillus | *Anoxybacillus flavithermus* | OM842900 |
| 59 | FB4_71 | Fumarole Bay | 100ºC | 100 | Bacteria; Firmicutes; Bacilli; Bacillales; Bacillaceae; Anoxybacillus | *Anoxybacillus flavithermus* | OM842901 |
| 60 | FB4_72 | Fumarole Bay | 100ºC | 100 | Bacteria; Firmicutes; Bacilli; Bacillales; Bacillaceae; Anoxybacillus | *Anoxybacillus flavithermus* | OM842902 |
| 61 | FB4_73 | Fumarole Bay | 100ºC | 100 | Bacteria; Firmicutes; Bacilli; Bacillales; Bacillaceae; Anoxybacillus | *Anoxybacillus flavithermus* | OM842903 |
| 62 | FB4_74 | Fumarole Bay | 100ºC | 99 | Bacteria; Firmicutes; Bacilli; Bacillales; Bacillaceae; Brevibacillus | *Brevibacillus thermoruber* | OM842904 |
| 63 | FB4_75 | Fumarole Bay | 100ºC | 100 | Bacteria; Firmicutes; Bacilli; Bacillales; Bacillaceae; Brevibacillus | *Brevibacillus* sp. | OM842905 |
| 64 | FB4_76 | Fumarole Bay | 100ºC | 99 | Bacteria; Firmicutes; Bacilli; Bacillales; Bacillaceae; Brevibacillus | *Brevibacillus thermoruber* | OM842906 |
| 65 | FB4_77 | Fumarole Bay | 100ºC | 100 | Bacteria; Firmicutes; Bacilli; Bacillales; Bacillaceae; Brevibacillus | *Brevibacillus thermoruber* | OM842907 |
| 66 | FB4_78 | Fumarole Bay | 100ºC | 100 | Bacteria; Firmicutes; Bacilli; Bacillales; Bacillaceae; Geobacillus | *Geobacillus stearothermophilus* | OM842908 |
| 67 | FB4_79 | Fumarole Bay | 100ºC | 99 | Bacteria; Firmicutes; Bacilli; Bacillales; Bacillaceae; Geobacillus | *Geobacillus* sp. | OM842909 |
| 68 | FB4_80 | Fumarole Bay | 100ºC | 100 | Bacteria; Firmicutes; Bacilli; Bacillales; Bacillaceae; Geobacillus | *Geobacillus stearothermophilus* | OM842910 |
| 69 | FB4_81 | Fumarole Bay | 100ºC | 100 | Bacteria; Firmicutes; Bacilli; Bacillales; Bacillaceae; Geobacillus | *Geobacillus stearothermophilus* | OM842911 |
| 70 | FB4_82 | Fumarole Bay | 100ºC | 100 | Bacteria; Firmicutes; Bacilli; Bacillales; Bacillaceae; Geobacillus | *Geobacillus* sp. | OM842912 |
| 71 | FB4_83 | Fumarole Bay | 100ºC | 99 | Bacteria; Firmicutes; Bacilli; Bacillales; Bacillaceae; Geobacillus | *Geobacillus* sp. | OM842913 |
| 72 | FB4_84 | Fumarole Bay | 100ºC | 99 | Bacteria; Firmicutes; Bacilli; Bacillales; Bacillaceae; Anoxybacillus | *Anoxybacillus tunisiense* | OM842914 |
| 73 | FB4_85 | Fumarole Bay | 100ºC | 100 | Bacteria; Firmicutes; Bacilli; Bacillales; Bacillaceae; Geobacillus | *Geobacillus stearothermophilus* | OM842915 |
| 74 | FB4_86 | Fumarole Bay | 100ºC | 98 | Bacteria; Firmicutes; Bacilli; Bacillales; Bacillaceae; Geobacillus | *Geobacillus* sp. | OM842916 |
| 75 | FB4_87 | Fumarole Bay | 100ºC | 100 | Bacteria; Firmicutes; Bacilli; Bacillales; Bacillaceae; Geobacillus | *Geobacillus* sp. | OM842917 |
| 76 | FB4_88 | Fumarole Bay | 100ºC | 100 | Bacteria; Firmicutes; Bacilli; Bacillales; Bacillaceae; Anoxybacillus | *Anoxybacillus* sp. | OM842918 |
| 77 | FB4_89 | Fumarole Bay | 100ºC | 100 | Bacteria; Firmicutes; Bacilli; Bacillales; Bacillaceae; Geobacillus | *Geobacillus kaustophilus* | OM842919 |
| 78 | FB4_90 | Fumarole Bay | 100ºC | 100 | Bacteria; Firmicutes; Bacilli; Bacillales; Bacillaceae; Geobacillus | *Geobacillus caldoxylosilyticus* | OM842920 |
| 79 | FB4_91 | Fumarole Bay | 100ºC | 100 | Bacteria; Firmicutes; Bacilli; Bacillales; Bacillaceae; Geobacillus | *Geobacillus caldoxylosilyticus* | OM842921 |
| 80 | FB4_92 | Fumarole Bay | 100ºC | 100 | Bacteria; Firmicutes; Bacilli; Bacillales; Bacillaceae; Geobacillus | *Geobacillus thermoleovorans* | OM842922 |
| 81 | FB4_93 | Fumarole Bay | 100ºC | 100 | Bacteria; Firmicutes; Bacilli; Bacillales; Bacillaceae; Geobacillus | *Geobacillus* sp. | OM842923 |
| 82 | FB4_94 | Fumarole Bay | 100ºC | 99 | Bacteria; Firmicutes; Bacilli; Bacillales; Bacillaceae; Geobacillus | *Geobacillus* sp. | OM842924 |
| 83 | FB4_95 | Fumarole Bay | 100ºC | 100 | Bacteria; Firmicutes; Bacilli; Bacillales; Bacillaceae; Geobacillus | *Geobacillus* sp. | OM842925 |
| 84 | FB4_96 | Fumarole Bay | 100ºC | 100 | Bacteria; Firmicutes; Bacilli; Bacillales; Bacillaceae; Geobacillus | *Geobacillus thermoleovorans* | OM842926 |
| 85 | FB4_97 | Fumarole Bay | 100ºC | 100 | Bacteria; Firmicutes; Bacilli; Bacillales; Bacillaceae; Geobacillus | *Geobacillus stearothermophilus* | OM842927 |
| 86 | FB4_99 | Fumarole Bay | 100ºC | 99 | Bacteria; Firmicutes; Bacilli; Bacillales; Bacillaceae; Geobacillus | *Geobacillus caldoxylosilyticus* | OM842928 |
| 87 | FB4_100 | Fumarole Bay | 100ºC | 100 | Bacteria; Firmicutes; Bacilli; Bacillales; Bacillaceae; Anoxybacillus | *Anoxybacillus flavithermus* | OM842929 |
| 88 | FB4_101 | Fumarole Bay | 100ºC | 100 | Bacteria; Firmicutes; Bacilli; Bacillales; Bacillaceae; Geobacillus | *Geobacillus caldoxylosilyticus* | OM842930 |
| 89 | FB4_102 | Fumarole Bay | 100ºC | 99 | Bacteria; Firmicutes; Bacilli; Bacillales; Bacillaceae; Geobacillus | *Geobacillus caldoxylosilyticus* | OM842931 |
| 90 | FB4_103 | Fumarole Bay | 100ºC | 99 | Bacteria; Firmicutes; Bacilli; Bacillales; Bacillaceae; Geobacillus | *Geobacillus jurassicus* | OM842932 |
| 91 | FB4_104 | Fumarole Bay | 100ºC | 100 | Bacteria; Firmicutes; Bacilli; Bacillales; Bacillaceae; Geobacillus | *Geobacillus* sp. | OM842933 |
| 92 | FB4_105 | Fumarole Bay | 100ºC | 100 | Bacteria; Firmicutes; Bacilli; Bacillales; Bacillaceae; Geobacillus | *Geobacillus* sp. | OM842934 |
| 93 | FB4_109 | Fumarole Bay | 100ºC | 100 | Bacteria; Firmicutes; Bacilli; Bacillales; Bacillaceae; Anoxybacillus | *Anoxybacillus flavithermus* | OM842935 |
| 94 | FB4_118 | Fumarole Bay | 100ºC | 98 | Bacteria; Firmicutes; Bacilli; Bacillales; Bacillaceae; Geobacillus | *Geobacillus* sp. | OM842936 |
| 95 | WB1_122 | Whalers Bay | 50ºC | 100 | Bacteria; Firmicutes; Bacilli; Bacillales; Bacillaceae; Geobacillus | *Geobacillus caldoxylosilyticus* | OM842937 |
| 96 | WB1_123 | Whalers Bay | 50ºC | 100 | Bacteria; Firmicutes; Bacilli; Bacillales; Bacillaceae; Anoxybacillus | *Anoxybacillus flavithermus* | OM842938 |
| 97 | WB1_124 | Whalers Bay | 50ºC | 100 | Bacteria; Firmicutes; Bacilli; Bacillales; Bacillaceae; Anoxybacillus | *Anoxybacillus flavithermus* | OM842939 |
| 98 | WB1_125 | Whalers Bay | 50ºC | 100 | Bacteria; Firmicutes; Bacilli; Bacillales; Bacillaceae; Anoxybacillus | *Anoxybacillus mongoliensis* | OM842940 |
| 99 | WB1_126 | Whalers Bay | 50ºC | 100 | Bacteria; Firmicutes; Bacilli; Bacillales; Bacillaceae; Anoxybacillus | *Anoxybacillus flavithermus* | OM842941 |
| 100 | WB1_127 | Whalers Bay | 50ºC | 99 | Bacteria; Firmicutes; Bacilli; Bacillales; Bacillaceae; Anoxybacillus | *Anoxybacillus tunisiense* | OM842942 |
| 101 | WB1_128 | Whalers Bay | 50ºC | 100 | Bacteria; Firmicutes; Bacilli; Bacillales; Bacillaceae; Anoxybacillus | *Anoxybacillus flavithermus* | OM842943 |
| 102 | WB1_129 | Whalers Bay | 50ºC | 99 | Bacteria; Firmicutes; Bacilli; Bacillales; Bacillaceae; Anoxybacillus | *Anoxybacillus tunisiense* | OM842944 |
| 103 | WB1_131 | Whalers Bay | 50ºC | 100 | Bacteria; Firmicutes; Bacilli; Bacillales; Bacillaceae; Brevibacillus | *Brevibacillus thermoruber* | OM842945 |
| 104 | WB1_132 | Whalers Bay | 50ºC | 100 | Bacteria; Firmicutes; Bacilli; Bacillales; Bacillaceae; Brevibacillus | *Brevibacillus thermoruber* | OM842946 |
| 105 | WB1_133 | Whalers Bay | 50ºC | 99 | Bacteria; Firmicutes; Bacilli; Bacillales; Bacillaceae; Brevibacillus | *Brevibacillus thermoruber* | OM842947 |
| 106 | WB1_134 | Whalers Bay | 50ºC | 100 | Bacteria; Firmicutes; Bacilli; Bacillales; Bacillaceae; Brevibacillus | *Brevibacillus thermoruber* | OM842948 |
| 107 | WB1_135 | Whalers Bay | 50ºC | 100 | Bacteria; Firmicutes; Bacilli; Bacillales; Bacillaceae; Brevibacillus | *Brevibacillus thermoruber* | OM842949 |
| 108 | WB1_136 | Whalers Bay | 50ºC | 98 | Bacteria; Firmicutes; Bacilli; Bacillales; Bacillaceae; Brevibacillus | *Brevibacillus thermoruber* | OM842950 |
| 109 | WB1_137 | Whalers Bay | 50ºC | 94 | Bacteria; Firmicutes; Bacilli; Bacillales; Bacillaceae; Brevibacillus | *Brevibacillus thermoruber* | OM842951 |
| 110 | WB1_138 | Whalers Bay | 50ºC | 100 | Bacteria; Firmicutes; Bacilli; Bacillales; Bacillaceae; Geobacillus | *Geobacillus* sp. | OM842952 |
| 111 | WB2_142 | Whalers Bay | 60ºC | 100 | Bacteria; Firmicutes; Bacilli; Bacillales; Bacillaceae; Anoxybacillus | *Anoxybacillus mongoliensis* | OM842953 |
| 112 | WB2_143 | Whalers Bay | 60ºC | 100 | Bacteria; Firmicutes; Bacilli; Bacillales; Bacillaceae; Anoxybacillus | *Anoxybacillus* sp. | OM842954 |
| 113 | WB2_144 | Whalers Bay | 60ºC | 100 | Bacteria; Firmicutes; Bacilli; Bacillales; Bacillaceae; Brevibacillus | *Brevibacillus thermoruber* | OM842955 |
| 114 | WB2_145 | Whalers Bay | 60ºC | 100 | Bacteria; Firmicutes; Bacilli; Bacillales; Bacillaceae; Brevibacillus | *Brevibacillus thermoruber* | OM842956 |
| 115 | WB2_146 | Whalers Bay | 60ºC | 100 | Bacteria; Firmicutes; Bacilli; Bacillales; Bacillaceae; Brevibacillus | *Brevibacillus thermoruber* | OM842957 |
| 116 | WB2_147 | Whalers Bay | 60ºC | 99 | Bacteria; Firmicutes; Bacilli; Bacillales; Bacillaceae; Brevibacillus | *Brevibacillus thermoruber* | OM842958 |
| 117 | WB2_148 | Whalers Bay | 60ºC | 99 | Bacteria; Firmicutes; Bacilli; Bacillales; Bacillaceae; Brevibacillus | *Brevibacillus thermoruber* | OM842959 |
| 118 | WB2_149 | Whalers Bay | 60ºC | 100 | Bacteria; Firmicutes; Bacilli; Bacillales; Bacillaceae; Brevibacillus | *Brevibacillus thermoruber* | OM842960 |
| 119 | WB2_150 | Whalers Bay | 60ºC | 100 | Bacteria; Firmicutes; Bacilli; Bacillales; Bacillaceae; Brevibacillus | *Brevibacillus thermoruber* | OM842961 |
| 120 | WB2_151 | Whalers Bay | 60ºC | 99 | Bacteria; Firmicutes; Bacilli; Bacillales; Bacillaceae; Geobacillus | *Geobacillus stearothermophilus* | OM842962 |
| 121 | WB2_152 | Whalers Bay | 60ºC | 99 | Bacteria; Firmicutes; Bacilli; Bacillales; Bacillaceae; Geobacillus | *Geobacillus stearothermophilus* | OM842963 |
| 122 | WB2_153 | Whalers Bay | 60ºC | 100 | Bacteria; Firmicutes; Bacilli; Bacillales; Bacillaceae; Geobacillus | *Geobacillus stearothermophilus* | OM842964 |
| 123 | WB2_154 | Whalers Bay | 60ºC | 99 | Bacteria; Firmicutes; Bacilli; Bacillales; Bacillaceae; Geobacillus | *Geobacillus stearothermophilus* | OM842965 |
| 124 | WB2_155 | Whalers Bay | 60ºC | 100 | Bacteria; Firmicutes; Bacilli; Bacillales; Bacillaceae; Geobacillus | *Geobacillus* sp. | OM842966 |
| 125 | WB2_157 | Whalers Bay | 60ºC | 97 | Bacteria; Firmicutes; Bacilli; Bacillales; Bacillaceae; Anoxybacillus | *Anoxybacillus* sp. | OM842967 |
| 126 | WB2_159 | Whalers Bay | 60ºC | 97 | Bacteria; Firmicutes; Bacilli; Bacillales; Bacillaceae; Anoxybacillus | *Anoxybacillus* sp. | OM842968 |

**Supplementary Table 3** Average Nucleotide Identity based on Blast (ANIb) for the genomes analyzed.

|  | **Strain FB4_88** | ***Geobacillus* sp. 12AMOR1** | ***Anoxybacillus* sp. B7M1** | ***Anoxybacillus amylolyticus* DSM 15939 [T]** | ***Anoxybacillus flavithermus* NBRC 109594** | ***Anoxybacillus flavithermus* TNO-09.006** | ***Anoxybacillus flavithermus* WK1** |
| --- | --- | --- | --- | --- | --- | --- | --- |
| **Strain FB4_88** | * | 72.37 | 72.68 | 74.6 | 95.76 | 92.43 | 94.07 |
| ***Geobacillus* sp. 12AMOR1** | 73.16 | * | 72.06 | 73.22 | 72.7 | 73.18 | 73.16 |
| ***Anoxybacillus* sp. B7M1** | 72.62 | 71.8 | * | 75.61 | 73.15 | 72.7 | 72.95 |
| ***Anoxybacillus amylolyticus* DSM 15939 [T]** | 74.64 | 73.09 | 75.94 | * | 74.59 | 74.87 | 75.11 |
| ***Anoxybacillus flavithermus* NBRC 109594** | 96.17 | 71.89 | 73.33 | 74.64 | * | 92.21 | 94.72 |
| ***Anoxybacillus flavithermus* TNO-09.006** | 92.79 | 72.5 | 73.16 | 75.16 | 92.43 | * | 91.64 |
| ***Anoxybacillus flavithermus* WK1** | 94.6 | 71.94 | 73.45 | 75.28 | 94.79 | 91.54 | * |
